# Metabolic engineering of cottonseed oil biosynthesis pathway via RNA interference

**DOI:** 10.1038/srep33342

**Published:** 2016-09-13

**Authors:** Zhongping Xu, Jingwen Li, Xiaoping Guo, Shuangxia Jin, Xianlong Zhang

**Affiliations:** 1 National Key Laboratory of Crop Genetic Improvement, Huazhong Agricultural University, Shizishan Street, Wuhan, Hubei 430070, China

## Abstract

Cottonseed oil is recognized as an important oil in food industry for its unique characters: low flavor reversion and the high level of antioxidants (VitaminE) as well as unsaturated fatty acid. However, the cottonseed oil content of cultivated cotton (*Gossypium hirsutum*) is only around 20%. In this study, we modified the accumulation of oils by the down-regulation of phosphoenolpyruvate carboxylase 1 (*GhPEPC1*) via RNA interference in transgenic cotton plants. The qRT-PCR and enzyme activity assay revealed that the transcription and expression of *GhPEPC1* was dramatically down-regulated in transgenic lines. Consequently, the cottonseed oil content in several transgenic lines showed a significant (P < 0.01) increase (up to 16.7%) without obvious phenotypic changes under filed condition when compared to the control plants. In order to elucidate the molecular mechanism of *GhPEPC1* in the regulation of seed oil content, we quantified the expression of the carbon metabolism related genes of transgenic *GhPEPC1* RNAi lines by transcriptome analysis. This analysis revealed the decrease of *GhPEPC1* expression led to the increase expression of triacylglycerol biosynthesis-related genes, which eventually contributed to the lipid biosynthesis in cotton. This result provides a valuable information for cottonseed oil biosynthesis pathway and shows the potential of creating high cottonseed oil germplasm by RNAi strategy for cotton breeding.

Allotetraploid upland cotton (*Gossypium hirsutum* L.) is one of the widespread cultivation domesticated genera of Gossypium in the family of Malvaceae. Its cultivation can be dated from as long ago as 3,500^1^ BC and is supposed to be ideal fiber crop compared to other three cultivars due to unparalleled yield and improved fiber productivity. With the completion of allotetraploid *G. hirsutum*^2^,^3^ and *G. babardence*^4^ genome sequencing, combined with *G. raimondii* (DD; 2n = 26)^5^ and *G. arboreum* (AA; 2n = 26)^6^, these genome sequencing sheds new light on unraveling cotton oil biosynthesis pathway and others basic metabolism in cotton plants.

Although cotton fiber is widely recognized as the main production of cotton cultivation. The cottonseed is the world’s sixth largest source of vegetable oil^7^. At the same time, cottonseed oil is recognized as beneficial for the health and has been widely used for deep frying of snack foods and food service for its main merit: low flavor reversion especially when used for cooking at high temperatures. Cottonseed oil produced by traditional upland cotton is of relatively high quality, which typically consists of 71% unsaturated fatty acid: 13% oleic acid (18:1) and 58% linoleic acid (18:2)^8^, almost comparable with oleic 39.6% and linoleic 46.0% in sesame^9^. Notably, the cottonseeds contain one of highest levels of total tocopherol from 13 different plant and animal sources of fats and oils including corn, soybean, sunflower, sesame, rapeseeds and palm oils^10^.

The plant oil is largely stored in seed in the form of oil droplets, which interacts with oleosin to maintain its stability in the cotyledons cells, and is consumed during the initial stage of seed germination^11^. Due to the increase in consumption of cottonseed oil and the bottleneck of the natural production, it is vital to create the high oil germplasm resources for cotton breeding by metabolic engineering. Weselake *et al*. systematically summarized the effectiveness of canola and soybean oil content improvement by genetic engineering, which involved in the over-expression of triacylglycerol (TAG) biosynthetic enzymes as well as modification of the upstream carbon flow, and transcription factors (TFs) that regulate the expression of multiple genes, and provide a good reference for us to enhance the contents of cottonseed oil^12^. A dominant low-seed-oil mutant (lo15571) of *Arabidopsis* was generated by the enhancer tagging system as well as a protein synthesis-related gene *At1g0150* was identified by Meyer and his colleagues^13^. Due to the silence of this *At1g01050* gene by seed-preferred RNA interference, increase in seed oil content was observed from 1% to 4%, mostly at the expense of seed storage protein. This study demonstrates the important role of pyrophosphate in regulation of carbon flow in oil and protein synthesis and also provide a novel route to modify seed composition by biotechnology.

Phosphoenolpyruvate carboxylase (PEPC, EC 4.1.1.31, PDB ID: 3ZGE) and Acetyl-CoA carboxylase (ACC, EC 6.4.1.2) are generally considered to play critical role in the regulation of assimilate carbon flow into oil and protein biosynthesis pathway (Fig. 1). ACC is a biotin-dependent multi-subunit enzyme that catalyzes the ‘irreversible’ carboxylation of acetyl-CoA to produce malonyl-CoA through ATP to provide energy^14^. As a result, malonyl-CoA substrate enters into the fatty acids biosynthesis pathway (Fig. 1). Whereas, PEPC, a carboxy-lyases enzyme that catalyses the ‘irreversible’ β-carboxylation of phosphoenolpyruvate (PEP) to oxaloacetate (OAA) and Pi (inorganic phosphate)^15^. OAA and Pi conjugate with acetyl-CoA to generate citric acid and enter into tricarboxylic acid cycle (TCA) to provide energy as well as other intermediate metabolites for life activities including a variety of intermediates required for the proteins biosynthesis. It had been observed in 1989 that the PEPCase activity was positively correlated with protein content and inversely correlated with lipid contents in soybean cultivar^16^. However, it was not experimentally confirmed until the experiments by Deng *et al*. proved the role of PEPCase in the accumulation of protein and oil through RNAi/over-expression of *CrCIS* and *CrPEPC1* genes in *C. reinhardtii*. In addition, they investigated the carbon source used for biosynthesis of the fatty acids only accounted for 45.9% of the total amount of photosynthetic carbon fixation^17^,^18^. Therefore, it has been proposed that due to the higher activity of PEPCase, a large number of carbon assimilation would be consumed in the TCA pathway rather than storage in seed oil.

RNAi (RNA interference), a post transcriptional gene silencing (PTGS) mechanism occurs in the cell’s cytoplasm, which is controlled by the RNA-induced silencing complex (RISC) and initiated by short double-stranded RNA (dsRNA)^19^. It has been developed as an efficient gene-silencing strategy in functional genomics, therapeutic intervention, agriculture and other areas since first discovered in *Caenorhabditis elegans*^20^,^21^,^22^,^23^. Therefore, in this report, an optional strategy for cottonseed oil content modification was proposed, by down-regulation of PEPC expression through RNAi strategy, allowing more flow of the carbon into the fatty acid biosynthesis pathway than into protein biosynthesis pathway.

Although the current research of PEPC is mainly concentrated in photosynthesis, still there are several reports which focus on the PEPC’s function on carbon flux distribution. A 14–28% increase in oil content of *C. reinhardtii* was observed during the down-regulation of *pepc2* gene by RNAi suggesting a negative correlation between *pepc2* mRNA abundance and oil content^24^. Down-regulation of *CrPEPC1* gene in *C. reinhardtii* by RNAi decreased PEPC activities by 39–50% and subsequently increased TAG level by 20%. Moreover, the decrease in *CrPEPC1* expression was observed to increase the expression of TAG biosynthesis-related genes. Conversely, *CrPEPC1* over-expression decreased TAG level by 37% and increased PEPC activities by 157–184%. These observations suggest that the oil content of algal cells can be controlled by regulation of the *CrPEPC1* gene expression^17^. Another important gene in carbon metabolic pathway- *Chlamydomonas citrate synthase* (*CrCIS*) was also investigated by the same team. The results showed that the down-regulation of *CrCIS* gene by RNAi could also increase the lipid content of algal cells. In the algal cells, the TAG level was increased by 169.5% after decreasing of *CrCIS* activity by 16.7% to 37.7% in the transgenic algae. Moreover, the decrease in *CrCIS* expression led to the increase in expression of TAG biosynthesis-related genes^18^. In Arabidopsis, the *PEPC* gene was found to be a crucial gene having role in modulating the balance of carbon and nitrogen metabolism. The transgenic plants accumulated more starch and sucrose but significantly lower nitrate levels were observed during the performance of double knockout of ppc1/ppc2^25^.

The first cotton *PEPC* cDNAs was cloned by RACE with the length of 3405 bp, but the detailed analysis about its function was not reported^26^. A new phosphoenolpyruvate carboxylase gene *GhPEPC2* was isolated from cotton (*G. hirsutum* cv. zhongmian 35) by RACE-PCR as well as its genetic information, expression patterns and catalytic were also described^27^. Li *et al*. examined the molecular basis of GhPEPC1 and 2 by malate synthesis in cotton fiber rapid elongation phase^28^. Currently, any report about the function of PEPC in cotton carbon flow distribution is not reported.

However, the information of two PEPC isoforms, *GhPEPC1* (GenBank: AF008939.1) and *GhPEPC2* (GenBank: EU032328.1) is available in National Center for Biotechnology Information (NCBI). *GhPEPC2* is encoding 971 amino acids having molecular mass of 110.6 kD and pI of 5.56. The amino acids multiple sequence alignment revealed that this enzyme contain all the conserved functional domains. It has been shown by tissue expression analysis and function analysis that *GhPEPC2* might be a house-keeping gene, which might play important physiological roles in roots^27^. However, little information is available for *GhPEPC1* in cotton. In order to investigate the correlation between *GhPEPC* expression and oil accumulation in cotton, the *GhPEPC1* gene was down-regulated *GhPEPC1* in transgenic cotton plants by RNAi strategy. Our results revealed that cottonseed oil content is significantly increased in transgenic lines parallel with lower *GhPEPC1* transcription levels. Moreover, we quantified the expression of the carbon metabolism related genes of transgenic *GhPEPC1* RNAi lines and the control plants by transcriptome and biochemistry analysis, which revealed that the decrease in *GhPEPC1* expression led to the increase in expression of TAG biosynthesis related genes. Taken together, this report demonstrates the feasibility of improving cottonseed oil yield by the regulation of carbon flux through *GhPEPC* gene.

## Results

### Molecular analysis of *G. hirsutum* phosphoenolpyruvate carboxylase 1 (*GhPEPC1*) gene

The sequence analysis indicated that *GhPEPC1* encodes 965 amino acids having molecular weight of 110 kD and an isoelectric point of 5.95. Multiple sequence alignment of the PEPC amino acid sequence from *G. raimondii* genome^5^ and *G. hirsutum* in NCBI revealed that *GhPEPC1* contains a Ser residue in N terminus, which acts as a phosphorylation site and exclusively belongs to plant PEPCs^29^,^30^,^31^,^32^,^33^, and a Ala residue in C3 plant, different from C4 plant (Ser)^32^. Furthermore, *GhPEPC1* has all conserved motifs and function domains that had been identified in plant *PEPCs* gene family (Fig. S1)^28^,^32^. *GhPEPC1* contains total 47 phosphorylation sites, including 26 serine, 15 threonine and 6 tyrosine predicted by NetPhos2.0. Transmembrane helices, signal peptide and subcellular localization analysis showed that *GhPEPC1* might be a cytoplasmic solubility protein without transmembrane domains (Fig. S2a) and signal peptide (Fig. S2b). Furthermore, rooted phylogenetic analysis using the *PEPCs* coding sequence and *zea mays* (GI: 162461755) acting as outgroup showed two distinct branches, suggesting that *GhPEPC1* and *GhPEPC2* were belonged to two different branches (Fig. 2a) and might be discrepant in the terms of gene function. According to multiple alignment and motif search of *GhPEPC1* gene, finally we ascertained the conserved domain (*GhPPC*) in this gene as the RNAi target fragments (Fig. 2b).

### Silencing *GhPEPC1* resulted in significantly increased cotton seed oil

To determine the influences of *GhPEPC* transcriptional level on carbon flow to improve cottonseed oil accumulation, we constructed the conserved domain (*GhPPC*, 308 base pairs) fragments silencing vectors (Fig. 3a) of the *GhPEPC1* using special primers as described previously and investigated the effect of artificial silencing of *GhPEPC1* on oil content in *G. hirsutum* YZ-1. Enough positive transgenic lines were isolated after genetic transformation of cotton cultivar YZ-1 (Fig. S3), which were cultivated in greenhouses (T0 generation) and field (T1, T2 generation). The results of southern blot showed that T-DNA region of RNAi vector has been inserted into the upland cotton genome with single-copy (PPC8) or double-copies (PPC1) (Fig. S4).

The total cottonseed oil content of transgenic lines and null cotton plants were assayed by using the Nuclear Magnetic Resonance (NMR). Furthermore, Gas Chromatography was used to select some representative lines (according to the different fatty acid gradient in total cottonseed oil) for fatty acid composition assay. Determination of total cottonseed oil content in these transgenic lines and null plants showed that cottonseed oil content was significantly increased by 16.7% (p < 0.01) in GhPPC transgenic line-PPC1 (Fig. 3b) as compared to the control plants.

For fatty acid composition analysis, 0.2 mg/ml fatty acid standard solution (including Hexadecanoic acid, Heptadecanoic acid, Stearic acid, Oleic acid, Linoleic acid, Linolenic acid and Nonadecanoic acid) was used to determine the peak time of each fatty acid in the standard solution (Fig. S5). Then, the cottonseed fatty acid composition in different lines at different developmental stages were detected and the results show a relatively abundant linolenic acid content in leaves than cottonseed, but the linoleic acid is opposite. Another obvious trend is that the cottonseed begins a sharp oil accumulation during 10DPA to 30DPA. Hexadecanoic acid and linoleic acid, no doubt, are the major contributors in this process. Particularly, at maturation stage (40DPA), the cottonseed oil content was significantly increased (p < 0.05, F-test) in PPC1 transgenic line when compared to null plants (Fig. 4a,b), with linoleic acid increased by 39.3% and hexadecanoic acid by 37.1%.

With the increase of total cottonseed oil content, the total protein content in this cottonseed was found to be displayed downtrend (Table 1). A negative correlation between total oil and protein contents in cottonseed confirms the carbon competition in cottonseed. In our transgenic lines, more carbon was involved in the biosynthesis of oil rather than protein when RNAi the *GhPEPC1*.

### RNA-seq decrypted the negative effects of *GhPEPC 1* on fatty acid biosynthesis through regulating the carbon flow

To investigate the changes of transcript accumulation in RNAi transgenic lines, one of the transgenic lines PPC8 was used to construct the RNA-Seq library. A summary of read pairs and mapping information of this study were listed in Table S1. All the raw sequencing data were uploaded and published at the National Center for Biotechnology Information Sequence Read Archive (SRA) database with accession number SRP082303. Overall, we obtained a high reads mapped ratio, with an average of 76.37% clean reads mapped to the reference *G. hirsutum* genome^2^. The FPKM distribution and intercorrelations correlogram of all samples showed (Fig. S6a) a high correlation between the different biological repeats in same sample and lower correlation among the different samples, suggesting the RNA-seq results can be used for follow-up analysis. Notably, the correlation test showed an R^2^ = 0.95 similarity between transgenic line and null plants due to the almost identical genetic background except a transgenic event of the *GhPEPC1* gene silenced by RNAi (Fig. S6b). FPKM analysis showed a total of 82 DEGs between PPC8 transgenic line and null plants, of which 55 were up-regulated and 27 down-regulated. Subsequently, by comparison with the annotation result from Zhang’s report^2^ indicated that some of the genes identified here were novel and had not been annotated so far. The DEGs between the null plants and RNAi line was showed by using volcanic figure. The red dots are up-regulated genes and blue dots indicated down regulated genes (Fig. S7). Since we were concerned about the expression of all *GhPEPC* genes in transcriptional level, the line graph were performed to compare all *GhPEPCs* genes expression level (FPKM) between null and PPC8. Results revealed that only Gh_A09G0010 (*GhPEPC1*) gene expression level was significantly lower (p < 0.001, F-test) than in the null. Moreover, we also observed other *PEPC* homologous genes showing a slightly declined expression level (Fig. S8).

To further categorize the DEGs profiles, a hierarchical clustering analysis was performed with the FPKM of DEGs and visualized on a Heat map (Fig. 5). It has been revealed by functional enrichment analysis that the up-regulated DEGs were mainly involved in glycometabolism, energy transfer, amino acid transportation and photosynthesis, including chloroplast-related (Cluster 1), electron transport chain of photosynthesis (Cluster 2) and carbon fixation (Cluster 3) genes. One of the most strikingly up-regulated DEGs was ribulose-1,5-bisphosphate carboxylase (rbcl), which completes the carbon fixation of photosynthesis via catalyzing carboxylation of CO_2_ integrate into ribulose-1,5-bisphosphate and then produces 3-phosphoglycerate after hydrolysis^34^. Although the down-regulation of DEGs were relatively decentralized, several important genes, including glutamine-dependent asparagine synthase, were up-expressed as showing a high nitrogen/carbon ratio in chloroplasts for synthesizing asparagine for nitrogen transport and storage^35^. GO function categories for DEGs showed more genes function in diverse biological process such as metabolism and catalysis (Fig. S9), Blast2go enrichment analysis further proofed the projecting performance of RNAi effect of transgenic line to the *GhPEPC1* gene in carbon fixation and photosynthesis (Fig. 6). KEGG metabolic pathway analysis for these DEGs also revealed that most of these DEGs were focused on carbon metabolism, which included carbon fixation genes located in chloroplast and glycolysis and oxaloacetate metabolic related genes located in cytoplasmic matrix. The detailed metabolic processes to exhibit carbon flows and photosynthetic system to enhanced carbon absorption were summarized in Fig. S10. Taken together, all the data pointed out that down regulation of *GhPEPC1* resulting in the changes of gene expression level related to carbon metabolism, which directed more carbon source flowing into fatty acid biosynthesis.

### Decreased *GhPEPC1* mRNA abundance resulted in significant acetyl-CoA carboxylase increase in the transgenic RNAi lines

To confirm the transcription data of *GhPEPC1* in the transcriptome, PPC8 and PPC1 transgenic lines were analyzed for the mRNA abundance of *GhPEPC1* by qRT-PCR. The *GhPEPC1* mRNA transcription levels were dramatically decreased in transgenic lines (Fig. 7), suggesting the high silencing effect of *GhPEPC1* gene generated by RNAi strategy.

To decide whether the decreased *GhPEPC1* mRNA abundance drives more carbon sources flowing into fatty acid biosynthesis, we inspected the transcription of carbon flux corresponding genes based on the DEGs in RNA-seq and KEGG metabolic pathways analysis. The acetyl-CoA carboxylase biotin carboxyl carrier protein (ACC) gene was analyzed by qRT-PCR. Increase in ACC transcription levels was observed in transgenic plant compared with null plant. Most importantly, the ACC expression levels and the content of cottonseed oil in different lines were positively related (R^2^ = 0.66) and the expression level of ACC gene was significantly (Fisher’s exact test, P value < 0.05) increased in PPC1 lines compared with null plant (Fig. 7).

### Down-regulation of *GhPEPC1* resulted in declined PEPCase enzyme activity, lower pyruvate and oxaloacetate content

PEPC usually catalyzes PEP to OAA. The OAA is a major metabolic intermediate in many processes that occur in plants including citric acid cycle, amino acid and fatty acid synthesis. However, upland cotton has complex genomes and also contained multiple copies of *GhPEPC* genes. Thus, does one gene silencing among all the *PEPC* genes affect the PEPCase enzyme activity? To answer this question, we performed the assay of PEPCase enzyme activity. The results showed that RNAi transgenic lines had significantly lower the enzyme activity (Fig. 8), which only account for 42.6–52.1% of the null plants (p < 0.05, F-test). As affected by declined PEPCase enzyme activity, the content of direct product the ATP– dependent carboxylation -OAA catalyzed by PEPCase enzyme was also found to be decreased down to 62.0% (Fig. 8). The substrate content of PEPCase –pyruvate presented obvious variation. The pyruvate content was significantly declined (23.0% lower than the null plants) in transgenic lines PPC1 with higher fatty acid content increases (16.7% higher than the null plants) (Fig. 8). Combining the lower pyruvate content and higher expression level of ACC (Fig. 7) together, we can speculate that acetyl CoA could not fully enter TCA because of the severe deficiency of oxaloacetate, and then the excess acetyl CoA was transported into the plastid and involved into fatty acid biosynthesis via ACC catalysis.

### Down-regulation of *GhPEPC1* did not disturb the plant photosynthesis and development of transgenic lines under the field conditions

It is well known that phosphoenolpyruvate carboxylase is a key enzyme involved in photosynthesis. So we evaluated the phenotype and major agronomic traits of transgenic descendants under field conditions at three research station located in Hubei province-Ezhou, Wuhan and Campus station from May 1st to October 1st in several major parameters of photosynthesis (photosynthetic rate, stomatal conductance and transpiration rate), chlorophyll and other agronomic traits of transgenic lines were investigated to evaluate the effects of RNAi silencing *GhPEPC1* gene on cotton growth and yield. All the transgenic plants did not show obvious phenotype changes compared with the null plants at three filed condition tests (Fig. S12a).

As shown in Table S3, chlorophyll content were all slightly declined for RNAi lines in different research stations and statistics analysis revealed that this difference was significant in some lines (p < 0.05, F-test). Decrease in photosynthesis was observed in transgenic plants as comparsion with null plants (Fig. S11a). To investigate the circadian rhythm changes of photosynthesis, we measured photosynthetic rate at three time points within the same day. The results revealed that photosynthetic rate intensity of transgenic lines and null plants showed similar trends: followed by morning >noon >afternoon. The proper order of stomatal conductance and transpiration rate was shown as follows: noon >morning >afternoon (Fig. S11b).

At the flowering stage of cotton plants, all the transgenic plants did not show obvious phenotype changes under field conditions (Fig. S12a,b). Interestingly, the size of cotton seeds was even enlarged when compared to the null cotton seeds (Fig. S12c). Boll number per plant is an important indicator to estimate the yield eventually. In this experiment, we found that the mean boll number per plant in most transgenic cotton lines were significantly higher than the control lines (Fig. 9a). Seed index (100-grain cottonseed weight) and cottonseed oil content between transgenic lines and null plants show slightly increased (Fig. 9b,c).

## Discussion

**Figure 1:**
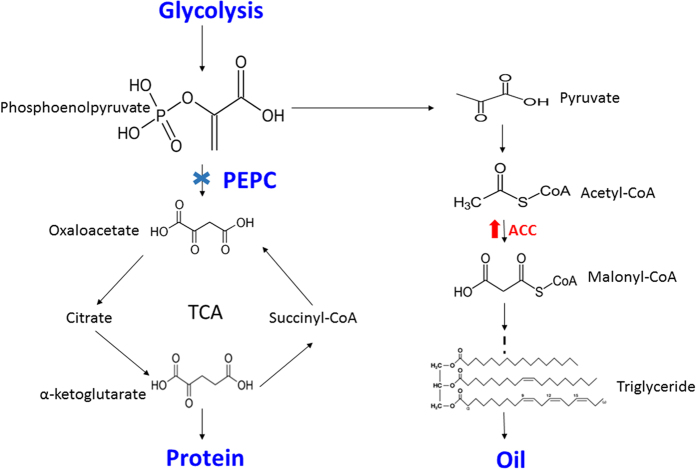
The schematic pathway for the distribution of carbon flow. PEPC (Phosphoenolpyruvate carboxylase) drives considerable carbon flux (Phosphoenolpyruvate) into TCA cycle and eventually control protein synthesis. Phosphoenolpyruvate is also the major substrate in the lipid biosynthesis pathway and ACC (Acetyl-CoA carboxylase) is the major enzyme that controls fatty acid biosynthesis. Since both protein and lipid biosynthesis pathway share the same substrate, the deceased consumption of phosphoenolpyruvate of either pathway is helpful to increase the final product (oil or protein) in the other pathway. In this report, the PEPC was downregulated via RNAi and theoretically decreased the carbon flow into the TCA cycle.

**Figure 2:**
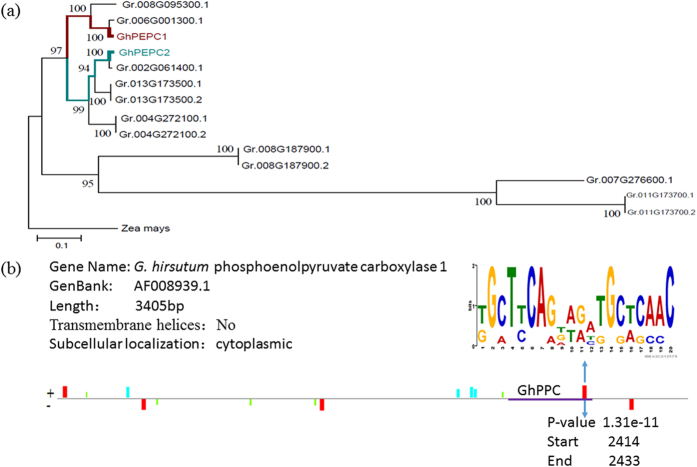
Phylogenetic analysis and comprehensive sequence information of *GhPEPC1* gene. (**a**) A neighbor-joining rooted phylogenetic analysis of *GhPEPCs* using *zea mays PEPC* as outgroup. (**b**) Sequence information of *GhPEPC1*, including the conserved domain (defined as *GhPPC*).

**Figure 3:**
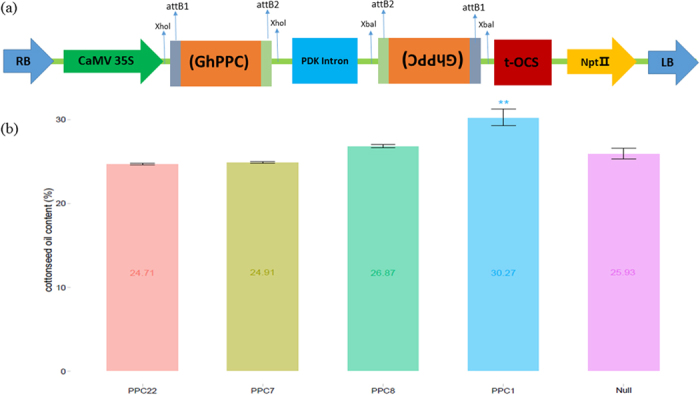
Schematic representation of T-DNA regions of the RNAi vectors and cottonseed oil content in T1 transgenic lines. (**a**) 35s-GhPPC RNAi vectors. (**b**) Cottonseed oil content in transgenic *GhPPC* lines and null plants; All the experiments were repeated three times. Bar graphs represent the average oil content ± SD of three replicates. *Denotes significant differences (P < 0.05).

**Figure 4:**
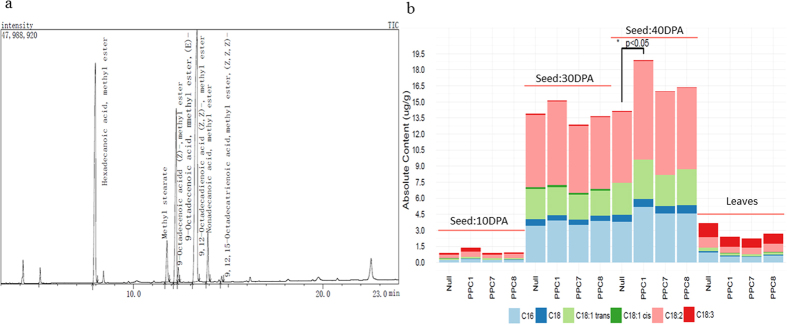
GC-MS determined the fatty acid composition of transgenic *GhPEPC1* lines and null plants. (**a**) GC-MS profile of fatty acid composition in 30DPA (days postanthesis) cottonseed. (**b**) Fatty acid profile in transgenic and null cottonseed and leaves at different development stages. Fisher’s exact test, *P value < 0.05.

**Figure 5:**
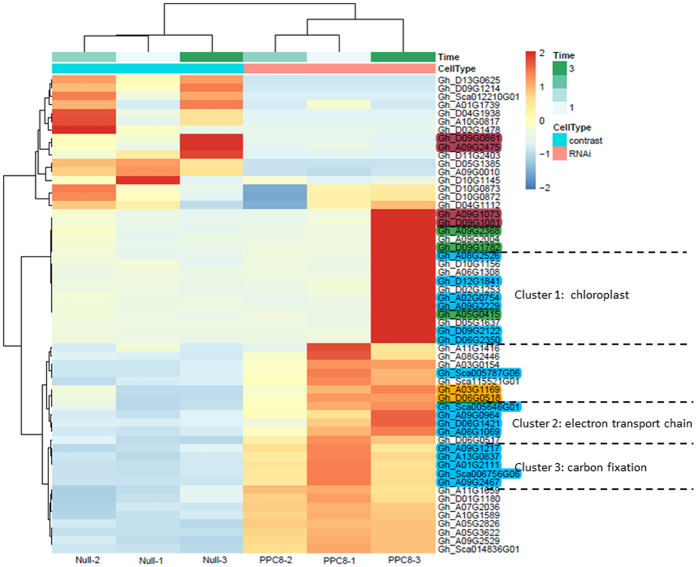
Heatmap show differential genes [log2 (FC) >2 and log2 (FC) <-2, FDR <0.05] clusters. Functional enrichment analysis revealed that the DEGs majorly involved in photosynthesis (ultramarine), glycometabolism (green), energy transfer (orange) and amino acid transportation (brown).

**Figure 6:**
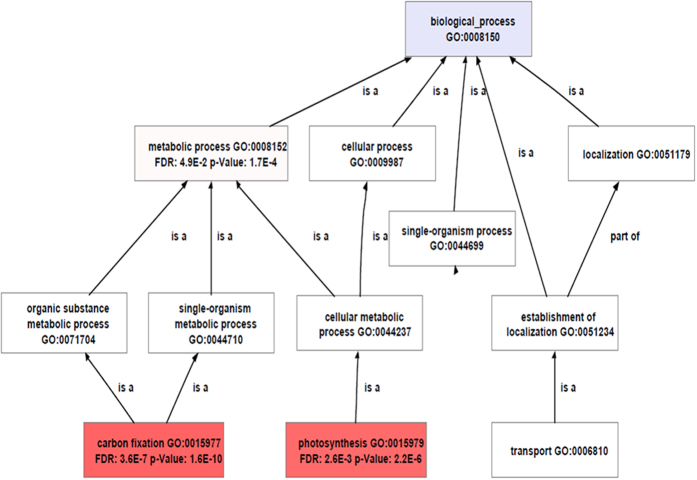
Gene ontology analysis of DEGs. GO enrichment analysis of biological process. These nodes are indicated with different colors proportionally according to their significance multiple-test P value (The value smaller, mean more significant and thus the nodes darker and redder), with the threshold FDR <0.05.

With the sharp increase of people’s living standards as well as frequent health threat related complications, such as obesity, cancer and cardiovascular disease, more and more people are concerned about healthy eating. Under this circumstance, more peoples fascinated cottonseed oil due to its high content of unsaturated fatty acid (linoleic acid), low content of trans-fatty acid, high level antioxidants (Vitamin E) and low flavor reversion. To meet this demand of increased consumption of cottonseeds oil, the metabolic engineering is recognized as a feasible and efficient strategy by changing the expression of key enzymes in metabolic process. Fortunately, many key genes involved in the metabolite biosynthesis especially for oil biosynthesis have been identified from diverse species. For example, an overexpression of the *Tropaeolum majus* diacylglycerol acyltransferase 1 (*TmDGAT1*), an enzyme that catalyzes the change of diacylglycerol combined with Acyl-CoA to triglycerides, resulted in an increase of 3.5–10% oil content in dry weight in wild-type *Arabidopsis* and confirmed the importance of serine/threonine site in this kinase^36^. Through detecting the level of acetyl-CoA, malonyl-CoA and free CoA using highly sensitive LC-MS/MS system, Avidan *et al*. proposed that more carbon flow into acetyl-CoA could improve the chloroplastic CoA pool level and higher acetyl-CoA biosynthesis could enhance TAG biosynthesis^37^. In the case of nitrogen (N) deprivation in green algae *C. reinhardtii*, the comprehensive study of PEPC^24^ and ACC^37^ genes confirmed the key roles of both genes in the regulation of carbon flow distribution (Fig. 1). This research is also highly consistent with our proposal that more carbon flow would enter into fatty acid biosynthesis by down-regulation of the *GhPEPC1* gene via RNAi strategy.

Compared to a single function gene, more researchers are paying attention to the transcription factor due to its ability to regulate multiple genes at the same time. An *et al*. expressed *AtWRI1* gene in the *Camelina sativa* and which increased the total seed oil by14% through the up-regulation of several genes related to the genes of lipid biosynthesis including pyruvate dehydrogenase E1α subunit, biotin carboxyl carrier protein and expansin 1^38^. In our report, the transcriptome data show that RNAi of *GhPEPC1* resulted in the expression level of several genes coinciding with existing reports, as well as expansin gene, which may involve in cell expansion^39^ to accommodate the increment of cottonseed oil content. Although *GhPEPC1* is not a transcription factor, this is a hub gene plays role in the basic metabolism net having close relationship with carbon flows. Consequently multiple related gene would be affected due to disturbance of expression of this gene.

**Figure 7:**
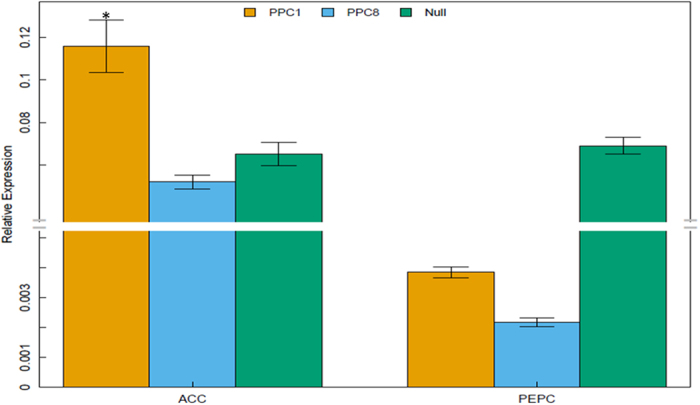
Relative expression level of *GhPEPC1* and other carbon flux distribution related genes. *GhPEPC1* and ACC genes relative expression levels in RNAi transgenic cotton lines and null were detected by qRT-PCR.

**Figure 8:**
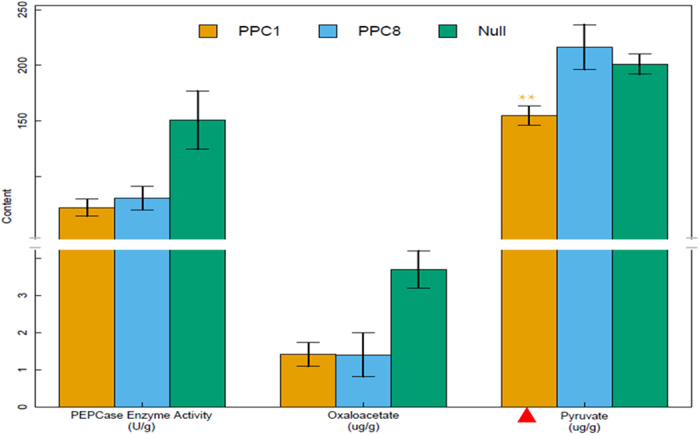
PEPCase enzyme activity, pyruvate and oxaloacetate content were detected in transgenic lines and null plants. The red triangle indicates that the plant has highest oil content in all the tested transgenic lines. All the experiments are repeated three times. Bar graphs represent the average activity or content ± SD of three replicates. *Denotes significant differences (P < 0.05).

**Figure 9:**
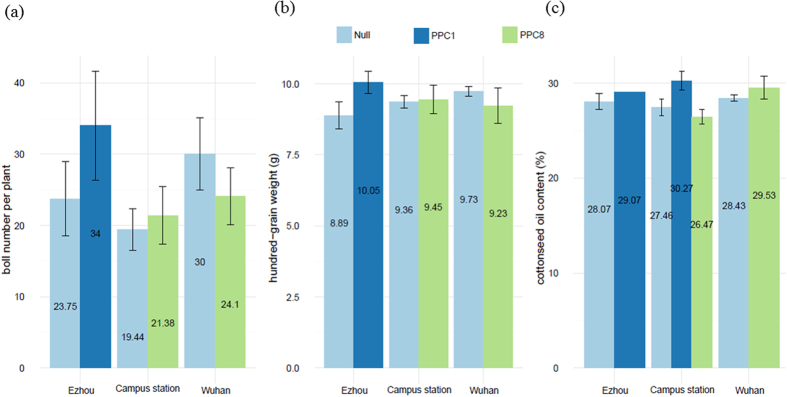
The agronomic traits of transgenic lines and null plants tested in the field condition. For boll number per plant, ten plants of each lines were selected for investigation. Three biological repeats were included for the analysis of 100-grain cottonseed weight and cottonseed oil content.

**Figure 10:**
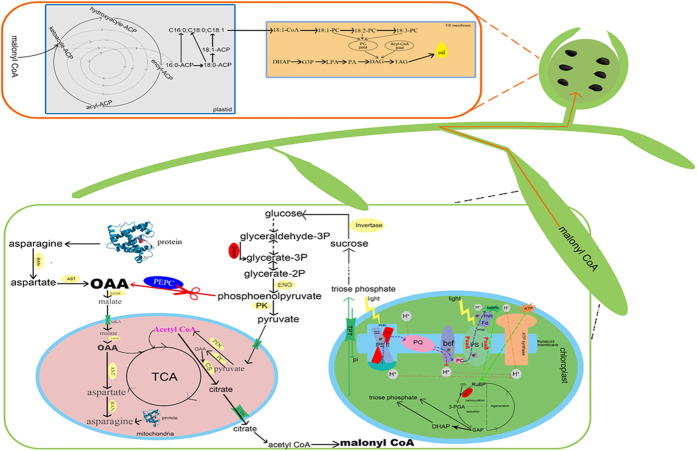
A proposal of the *GhPEPC 1* as a core enzyme to regulate carbon turnover and eventually improve seeds oil content in plant. *GhPEPC*1 works as a core enzyme not only involved in photosynthesis but also regulated the inflowing of carbon turnover to fatty acid biosynthesis and finally contributed to the increase of cottonseed oil content. In this report, the carboxylation pathway of PEP to OAA was blocked through RNAi of *GhPEPC1* and resulted in a decline in OAA concentration. Under this background, more proteins would be converted to aspartate involved in anaplerotic reactions to offset the OAA deficiency in mitochondrial and this hypothesis has been confirmed by our RNA-seq data. Among the DEGs, the glutamine-dependent asparagine synthase 1 was found to be down-regulated in the RNAi lines. Simultaneously, more pyruvate will be transported into mitochondria due to the acceleration of glycolysis, through mitochondrial pyruvate carrier (MPC) located in the mitochondrial inner membrane. The pyruvate located in mitochondria was then involved into two metabolism branches: conversion into acetyl-CoA through pyruvate decarboxylation with pyruvate dehydrogenase complex (PDC) and other irreversible carboxylation to form OAA by pyruvate carboxylase (PC) ligase to serves as an anaplerotic reaction for TCA. The excessive acetyl CoA and relative lack of OAA forced chloroplasts to the heighten light-dependent reactions based on photosynthetic electron transport chains and which produced the ATP and NADPH by using Calvin cycle, where the fixed CO2 was converted as sucrose to provide substrate for glycolysis. However, the RNAi cotton plant was in a state of ‘starvation’ because of the down-regulation of *GhPEPC* and the TCA were confined. Moreover, the expression levels of ACC in transgenic lines were significantly increased, which indicates that superfluous acetyl-CoA could combine with OAA and form citrate and then transported to cytoplasm via citrate transport protein (CTP). These citrates have participated into biosynthesis of fatty acids and finally stored in cottonseed in the form of TAG. The red marker region indicated that relevant genes exhibiting rising trend in RNA-seq data. The blue marker indicated down-regulated genes.

Based on previous research and our data, we proposed a working model to integrate the metabolic regulation mechanism of *GhPEPC1* with regard to the carbon and lipid metabolic in cotton. *GhPEPC*1 works as a core enzyme not only involved in photosynthesis but also regulated the inflowing of carbon turnover to fatty acid biosynthesis and finally contributed to the increase of cottonseed oil content (Fig. 10). The TCA was initiated when citrate synthase catalyzed the condensation reaction of mitochondria OAA and acetyl-CoA to citrate. Furthermore, the TCA as a hub metabolism is one of the most important cycles for plant life because this cycle generated major energy and provided carbon skeleton for the biosynthesis of other substances, such as fatty acid and porphyrin ring. But this process was strongly affected by OAA concentration in mitochondria due to more consumption of OAA in other biosynthetic reactions. Therefore, several special OAA anaplerotic reactions are necessary, which includes the carboxylation of pyruvate and PEP and transamination of aspartate, existing in mitochondria for the conversion of OAA to TCA. In this report, the carboxylation pathway of PEP to OAA was blocked through RNAi of *GhPEPC1*, an enzyme that catalyzes this conversion, and resulted in a decline in OAA concentration (Fig. 8). Under this background, more proteins would be converted to aspartate involved in anaplerotic reactions to offset the OAA deficiency in mitochondrial. Indeed, this hypothesis has been confirmed by our RNA-seq data. Among the DEGs, the glutamine-dependent asparagine synthase 1 was found to be down-regulated in the RNAi lines, which catalyzes the synthesis of asparagine-a major compound for nitrogen storage^35^. Simultaneously, more pyruvate will be transported into mitochondria due to the acceleration of glycolysis, through mitochondrial pyruvate carrier (MPC) located in the mitochondrial inner membrane (content of pyruvate was declined in our experimental results (Fig. 8)). The pyruvate located in mitochondria was then involved into two metabolism branches: the conversion into acetyl-CoA through pyruvate decarboxylation with pyruvate dehydrogenase complex (PDC) and other irreversible carboxylation to form OAA by pyruvate carboxylase (PC) ligase to serves as an anaplerotic reaction for TCA (Fig. 10). The excessive acetyl CoA and relative lack of OAA forced chloroplasts to heighten the light-dependent reactions based on photosynthetic electron transport chains and which produced the ATP and NADPH by using Calvin cycle, where the fixed CO_2_ was converted as sucrose to provide substrate for glycolysis (data support from RNA-seq and Fig. 7). However, the RNAi cotton plant was in a state of ‘starvation’ because of the down-regulation of *GhPEPC* and the TCA were confined. Moreover, we found the expression levels of ACC in several transgenic lines were significantly increased when compared to null, which indicates that superfluous acetyl-CoA could combine with OAA and form citrate and then transported to cytoplasm via citrate transport protein (CTP). These citrates have participated into biosynthesis of fatty acids and finally stored in cottonseed in the form of TAG (Fig. 10).

In this metabolism engineering of cotton oil content, the *GhPEPC1* was selected as RNAi target gene and a number of transgenic lines were obtained through *Agrobacterium*-mediated genetic transformation by using 35S promoter. The results of genetic transformation presents two dilemma. On the one hand, Why does a few deleterious effects throughout the plant of down-regulating a very important gene by 35S promoter- mediated RNAi? For this question, as it is known that the upland cotton is allotetraploid and we found up to 31 copies of *GhPEPC* genes based on the *Gossypium hirsutum* genome^2^. So, the deleterious effects–resulted from 35s promoter-were maybe offset by some paralogs genes of RNAi target gene. On the other hand, we found only half of the transgenic lines showed significantly improve in oil content ranging from 5.0–16.7%. It has been speculated that one of the main reasons is that RNAi cannot thoroughly suppress the express of target genes. Luckily, we created a transgenic RNAi population with more than 40 independent lines and several lines such as PPC1 line showed obvious decrease in both transcription and activity of *GhPEPC1*. It is known that RNAi requires active repression effect on the target gene, but this effect could be weaken through increased target genes mRNA turnover and/or decrease RNA-induced silencing complex (RISC) turnover^40^. For this dilemma, CRISPR/Cas9, a genome accurate editing techniques depend upon dual tracrRNA:crRNA structure act as guide to direct Cas9 endonuclease to cleave the cognate target DNA^41^, may be the best alternative technology to knock down the target gene. Most importantly, CRISPR/Cas9 can be used to simultaneously inactive multiple genes in a single transformation step^42^,^43^,^44^. Indeed, it will be an efficient technology of two birds with one stone that produce large amounts of mutations with the target gene knockout at the same time. For further increase of the cotton oil content, we are currently constructing several CRISPR vectors targeting lipid biosynthesis related genes and would like to compare the down-regulation effects of RNAi and CRISPR system on the target gene.

In this report, it has also been found that most of DEGs identified by RNA-seq are less than 1kb in length (Fig. S13a). Under systematic study of human and mouse genomes Grishkevich and Itai proposed that gene length and expression levels are closely related with gene duplication and alternative splicing. Generally, short genes more tend to have low expression levels and few splice variants but many gene duplicates, it indicates that large gene families are less important for the development of plants^45^. If this hypothesis is right, it could partially explained that some transgenic RNAi lines did not show any significantly improvement in the cottonseed oil content. Because most DEGs identified from this line are short genes. In addition, these DEGs were split into four clusters based on phylogenetic analysis and many DEGs had relatively close genetic relationship and possibly divided from the same gene family, even exhibited functional redundancy (Fig. S13b).

In this study, we successfully modified the accumulation of oils by down-regulation of the *GhPEPC1* via RNAi in transgenic cotton plants. The results indicated that cottonseed oil content in transgenic plants showed a significantly increase up to 16.7% in comparison with control plants. The transcriptome analysis applied in transgenic and control plants revealed that most of DEGs were involved in the metabolism of carbon and lipid. This result provides valuable information for cotton oil biosynthesis pathway and shows the potential of creating high cottonseed oil content germplasm by RNAi strategy in cotton breeding.

## Materials and Methods

### RNA extraction

The total RNA of transgenic and null (negative offspring derived from genetic segregation of positive transgenic plant) cotton plants were prepared by using the modified guanidine thiocyanate method as previously described in our report^46^. Tissues were ground completely into powder by using mortar and pestle with liquid nitrogen and total RNAs were released from the cell with ice-cold RNA extraction buffer containing 1% β-mercaptoethanol. After a series of extraction and centrifugal precipitation, RNAs were precipitated by ice-cold 2 mol/L sodium acetate and isopropanol and then washed with 75% ethanol for twice. Finally, air-dried RNAs were dissolved in 30 μL diethylpyrocarbonate treated water. Spectrophotometer (NanoDrop 2000, Thermo scientific, USA) was used to determine the RNA concentration and agarose gel electrophoresis was used to measure its integrity.

### Sequence analysis of *GhPEPC1*

The primary structure of protein was predicted by using ExPASy-ProtParam tool (http://web.expasy.org/protparam/). Protein phosphorylation sites were performed by using NetPhos 2.0 (http://www.cbs.dtu.dk/services/NetPhos/). TMHMM (http://www.cbs.dtu.dk/services/TMHMM/) and SignalP (http://www.cbs.dtu.dk/services/SignalP/) were used to predict transmembrane helices and signal peptide. Protein subcellular localization was analyzed with PSORT II Prediction (http://psort.hgc.jp/form2.html), and the sequence alignments were completed using the DNAMAN and MEGA6 software with the neighbour-joining method for phylogenetic analysis.

### Construction of RNAi vector and cotton genetic transformation

Total RNA was isolated as mentioned above and the first strand of cDNA was synthesized by using the SuperScript III reverse transcriptase (Invitrogen, Carlsbad, CA, USA). Complete coding sequence *GhPEPC1* was obtained from NCBI (National Center for Biotechnology Information). The conserved domain and 3′-UTR-specific region fragments of the *GhPEPC1* gene were amplified respectively by PCR using primers GhPPC-F: 5′-TTGAATACTTCCGCCTAGCA -3′ and Gh PPC -R: 5′-AGCGATTCCAGGGTCTCC -3′. After purification of the DNA using Purification Kit (Gene Tech, Shanghai), the target gene fragments were inserted into RNAi vector pHellsgate 4 of the gateway system. The resulting plasmids was designated as 35s-GhPPC. All above DNA sequences analyzed were performed by Beijing Genomics Institute (BGI, Shenzhen, China). The expression constructs were introduced into the *Agrobacterium tumefaciens* strains EHA105 (kanamycin as selectable marker) after electroporation, and then used for genetic transformation via *Agrobacterium*-meditated in cotton cultivar YZ1 according to our previous publications^23^,^47^,^48^.

### DNA isolation and southern blot analysis

DNA was isolated from the young leaves of putative transgenic and null plants using a Plant Genomic DNA Kit (Tiangen Biotech, China). The 35S promoter forward primer from RNAi vector and target gene fragment reverse primer were used for PCR amplification of positive detection with transgenic lines.

Through the PCR amplification, the fragment of *NPTII* gene was used as a probe for Southern blotting to detect the copy number of transgene insertion. A DIG High Prime DNA Labeling and Detection Starter Kit II (Roche, USA) were used for Southern hybridization. First, the cotton genomic DNA was digested with *Hind* III -HF, electrophoresed on 0.8% agarose gel (DNA Molecular Weight Marker II labeled with DIG) and blotted onto a Hybond N+ membrane through salt bridge for hybridization. After hybridization and stringent washing, the Digoxigenin-11-dUTP was incorporated into the newly synthesized DNA probes via PCR amplification and then hybridized with the target DNA. Finally, a digoxin antibody that connected with alkaline phosphatase was used as detection probe labeled with digoxin. After completion of these protocols, CSDP (chemiluminescence substrate for alkaline phosphatase) was used to visualize the position of probe hybridization in x-rays.

### Cottonseed oil content and fatty acid profiling analysis

For determination of cottonseed oil content, cotton flowers were self on the first day postanthesis (DPA) and collected at four developmental stages, 10, 30, and 40 DPA, representing different stages of cottonseed oil accumulation in embryo or immature seeds according to previous report^11^. At each time point, ovules were isolated from developing cotton balls and stored at −80 °C for further oil content determination.

The method of Nuclear Magnetic Resonance (NMR) Spectroscopy (NIUMAG, Shanghai, China) was used to determine the mature oil content of cotton seed. The oil content of developing seeds was determined by using Gas Chromatography (GC) analyses as the Fatty Acid Mass Spectrometry Protocol^49^. From each developmental stage, immature seeds were ground into powder in a mortar under a liquid nitrogen environment, and then 0.1~0.2 g powder were added into 2 ml glass tubes for each biological replicates. For each sample, 5 biological replicates were included. Meantime, 1.5 mL of 2.5% sulfuric acid-methanol solution (chromatography methanol, concentrated sulfuric acid, butylated hydroxytoluene (BHT)), 200 μL C19-nonadecanoic acid (2 mg/ml) internal standard solution and 400 μL toluene were added to each tube and mixed. In order to completely melt the mixture of fatty acids, the samples were kept in water bath at 90 °C for 90 min. Following the incubation, 4 mL 0.9% NaCl and 500 μL hexane (HPLC grad) were added to each tube, and then mixed vigorously. Following the centrifugation (1000 rpm, 10 min), around 600 μL upper phase were aspirated and then the impurities were filtered out with 0.22 μm organic phase needle filter and transferred into vials for gas chromatography analyses.

The content of total methyl esters fatty acid composition was measured on a gas chromatograph-mass spectrometer (GCMS-QP2010 Ultra, Shimadzu, Japan). The oven temperature was maintained at 170 °C for 1 min and then increased in steps by 3 °C every min to 230 °C. Methyl ester derivatives were detected by using MS.

### Determination of cottonseed protein content

In consideration of the completed with protein and oil content in cottonseed, we were also determined the total protein content in mature cottonseed. Approximately 0.1 g cottonseed kernel was collected and mixed in 500 μL of protein extracting solution (20 mM Tris/Hcl, 100 mM NaCl, 20 mM KCl, 1.5 mM MgCl2, 0.5% igepal co-630, 0.5 mM PMSF, 5 mM EDTA, 1 mM DTT), then completely ground by using mortar and pestle. The mixture was transferred into 1.5 ml centrifuge tube and placed on ice for 5–10 min. After centrifugation at 13000 rpm at 4 °C for 15 min, the supernatant was pipetted and then again mixture was centrifuged to get final supernatant. Final total protein content was determined by using RC DC Protein Assay Kit (Bio-Rad, California, USA) following the standard assay protocol and calculated at the 750 nm absorbance within the standard curve, which BAS was used as standard protein.

### Transcriptome analysis for the transgenic plants by Illumina sequencing

Total RNA was extracted as previously described protocol. RNA samples, with qualified quality, were sequenced on the Illumina Hi-Seq 2500 sequencer (Illumina, SanDiego, CA) at the Biomarker Technologies (Beijing, China). All reads are available for download on NCBI bioproject (PRJNA339421). Clean reads were filtered from raw reads to remove low quality reads and adapter under this command parameters: LEADING:5 TRAILING:5 SLIDINGWINDOW:4:20 MINLEN:50 using Trimmomatic (http://www.usadellab.org/cms/). FastQC and SolexaQA quality visualization were applied and clean reads would be used for further analysis. RNA-seq analysis protocol contains sequence alignment, assembly, calculation of expression and mining of differentially expression genes (DEGs) referred Trapnell’s proposal^50^. TopHat with Bowtie selected as an alignment engine was used to map clean reads to the corresponding reference cotton genome *G. hirsutum* L. acc. TM-1^2^. The yielded alignment files were assembled and the expression level FPKM (fragments per kb per million reads) of each sample was calculated by using Cufflinks packages.

DEGs analysis was conducted using cuffdiff, a program of the Cufflinks packages, based on normalized FPKM read counts. For the results of cuffdiff, DEGs were captured based on absolute log_2_ fold change greater than 2 of each gene’s FPKM. The distribution of P-values was controlled for a false discovery rate by the BH method^51^ at α = 0.05 and q-value, which is a measure of false discovery rate (FDR) proposed by Storey^52^. Gene ontology (GO) enrichment analysis was performed using the Blast2GO and Web Gene Ontology Annotation Plot (WEGO)^53^. Filtered DEGs described previously were imported and carried out corresponding analysis under Fisher’s Exact Test and corrected for multiple testing with FDR < = 0.05. KOBAS 2.0 (KEGG Orthology Based Annotation System), which can identify statistically significantly enriched pathways from well-known pathway databases^54^, was used for metabolic pathways analysis tool for KEGG.

Visualization of RNA-seq data and DEGs in this report was performed by ggplot2 etc in R software v. 3.2.2 (R Project for Statistical Computing, Vienna, Austria). Hierarchical clustering of differentially expression genes were performed on FPKM with ward.D2 clustering method and visualized on Heat map in R using the pheatmap package.

### Quantitative real-time reverse transcription-PCR

Isolated total RNA of transgenic and null plants was reverse-transcribed to cDNA as previously described. Quantitative real-time PCR (qRT-PCR) was performed with the ABI Prism 7000 system (Applied Biosystems, Foster City, CA, USA) using SYBR Green as the fluorescent dye. In this qRT-PCR analysis, 3 plants of each transgenic line and null plants were randomly selected and sampled for each independent biological replicate. Gene sequences were obtained from the RNA-seq resulted and *G. hirsutum* coding sequence^2^. The primers are listed in Table S2. UBQ7 gene (GenBank: DQ116441.1) was used as a reference to normalize target genes expression values. Three technical replicates and Two-Step RT-PCR method were performed for each experiment. The relative quantification analysis was calculated by using 2 −ΔΔ*C*_T_. Error bars represent the standard deviation.

### Assay of PEPCase enzyme activity in transgenic and null plants

The PEPCase enzyme activity of transgenic lines and null plants were determined by using a phosphoenolpyruvate carboxylase kit (Comin, Suzhou, China) following the manufacturer’s instruction. Finally, one unit (U) of PEPCase activity was defined as the amount of PEPCase required to consume 1 nmol NADH of per gram tissue in per minute.

### Quantification of pyruvate and oxaloacetate in transgenic and control cotton plants

Pyruvate content was detected by using pyruvic acid assay kit (Comin, Suzhou, China). For the sample preparation, approximately 0.1 g leaf tissues were collected and mixed with 1 ml extracting solution, then rapidly homogenized on the ice. In order to fully extract pyruvate, the homogenate was quieted for 30 min and then centrifuged with 8000 × *g* for 10 min. Determination process was completed with the following steps: 300 μL supernatant and 100 μL 2,4-Dinitrophenylhydrazine assay buffer 1 were added into the 1.5 ml centrifuge tube. After well mixed and transitory quieted for 2 min, 500 μL alkaline assay buffer 2 was added and the absorbance were measured at 520 nm with L6 UV-visible spectrophotometer (INESA, shanghai, China). Final pyruvate content was calculated through the standard curve.

An oxaloacetate colorimetric assay kit (BioVision, CA, USA) was used to determine total oxaloacetate content in cotton following the recommended manufacturer’s instruction. Final OAA concentrations were also calculated based on the standard curve.

### Measurement of Photosynthesis and Chlorophyll under field condition

We designed filed test for T2 generation of transgenic population and null plants at three research stations, namely, Ezhou, Wuhan and Campus station. At the flowering stage, the photosynthesis rate of cotton plants was measured by using the LI-6400XT Portable Photosynthesis System (LI-COR^®^ Biosciences, Nebraska, USA) and chlorophyll contents were detected by SPAD 502 Plus Chlorophyll Meter (Spectrum Technologies, Aurora, USA ). The measurement time of photosynthesis was around 10:00 am, but for chlorophyll was inadvertent. In particular, we measured the photosynthesis at different time points in one of the research stations (Campus station) at 8 am, 2 pm and 5 pm, respectively.

### Determination of other agronomic traits under field condition

The cotton null plants, as well as transgenic lines derived from YZ1 were grown in the three different experiment stations as described previously under normal farming practices. At the end of September (the end of growth period of cotton in Huebei), the agronomic traits of all transgenic lines and null plants were determined including phenotypic traits and yield traits. For yield traits, seed index (100-grain cottonseed weight), cottonseed oil content and boll number per plant were determined.

## Conclusions

This is the first report of artificially improved oil content via RNAi strategy and the analysis of its metabolic mechanism in Upland cotton. Decreased *GhPEPC1* expression in transgenic cotton led to the increased expression of TAG biosynthesis related genes and elevated cottonseed oil content, which demonstrated the feasibility of improving cottonseed oil yield by regulating the carbon flux.

## Additional Information

**How to cite this article**: Xu, Z. *et al*. Metabolic engineering of cottonseed oil biosynthesis pathway via RNA interference. *Sci. Rep.* **6**, 33342; doi: 10.1038/srep33342 (2016).

## Supplementary Material

Supplementary Information

## Figures and Tables

**Table 1:** Mean content of oil and protein in moisture-free, delinted cottonseed.

Lines	Component content					
	Mean(%)	oil SD	P-value α = 0.05	Mean(mg/ml)	protein SD	P-value α = 0.05
PPC1	29.07	0.06	***	11.87	0.36	*
PPC8	27.36	0.1	*	13.11	0.79	—
Null	26.88	0.01	—	13.51	0.56	—
