# Supplementary material for: Metabolic engineering of cottonseed oil biosynthesis pathway via RNA interference
Source: Sci Rep. 2016 Sep 13;6:33342. doi: 10.1038/srep33342 (PMC5020431; doi:10.1038/srep33342)
Supplement: Supplementary Information [file srep33342-s1.pdf]

Supplementary information

Metabolic engineering of cottonseed oil biosynthesis pathway via RNA interference

Zhongping Xu, Jingwen Li, Xiaoping Guo, Shuangxia Jin* and Xianlong Zhang*

National Key Laboratory of Crop Genetic Improvement, College of Plant Science and Technology, Huazhong Agricultural University, Wuhan, Hubei,430070, China

* Corresponding author:

Shuangxia Jin and Xianlong Zhang

Fax: +86-027-8720196;

email: jsx@mail.hzau.edu.cn or xlzhang@mail.hzau.edu.cn

Zhongping Xu (tiramisutes@webmail.hzau.edu.cn)

Jingwen Li (hopetes@outlook.com)

Xiaoping Guo (xpguo@mail.hzau.edu.cn)

Shuangxia Jin (jsx@mail.hzau.edu.cn)

Xianlong Zhang (xlzhang@mail.hzau.edu.cn)

SUPPLEMENTARY FIGURE LEGEND

Fig. S1. Multiple sequence alignment of phosphoenolpyruvate carboxylase amino acid sequence from *Gossypium raimondii* genome and *Gossypium hirsutum* accession in NCBI. GhPEPC1 (GenBank: AF008939.1) and GhPEPC2 (GenBank: EU032328.1). Conserved motifs and function domains were annotated with red underline. *: C3 signature Ala; △: Phosphorylation site and exclusively belongs to plant PEPCs.

Fig. S2. Transmembrane helices (a) and signal peptide (b) of G. hirsutum phosphoenolpyruvate carboxylase 1 (*GhPEPC1*). The predicted resulted showed that *GhPEPC1* might be a cytoplasmic solubility protein for non-transmembrane domains (a) and non-signal peptide (b).

Fig. S3. **Agrobacterium-mediated genetic transformation of YZ1 with *GhPEPC1* gene.**

Fig. S4. **Southern blotting analysis of transgenic plants GhPPC.** DNA Molecular Weight Marker II labeled with DIG was acted as positive control.

Fig. S5. GC-MS from the analysis of fatty acid component for cottonseed and leaves. (a) GC-MS profiles of a standard solution. (b) GC-MS profile of a sample, which contained a 0.2mg/ml Nonadecanoic acid internal standard.

Fig. S6. **Correlation analysis.** (a) Intercorrelations correlogram of all sample. The darker and more saturated the color, the greater the magnitude of the correlation. The upper triangle of cells displays the same information using pies. (b) Replicates Correlation Test between contrast (Null) and RNAi (PPC8) based on FPKM value.

Fig. S7. **Volcanic figure showing the differentially expressed genes (DEGs) between the transgenic lines and null plants.** The black dots represent no difference, red dots are up-regulated genes and blue dots are down regulated genes.

Fig. S8. **The line graph show GhPEPC genes expression level (FPKM) between null (contrast) and PPC8 (RNAi).** Only Gh_A09G0010 (*GhPEPC1*) gene expression level was significantly lower than in the null plants (P value < 0.05).

Fig. S9. **Gene ontology analysis of DEGs.** (a) Functional classification of differentially expressed genes, according to Gene Ontology Consortium.

Fig. S10. Pathway of carbon metabolism from KEGG metabolic pathway analysis. Up red arrow indicated the relation genes were up-regulation in DEGs, down blue arrow were opposite.

Fig. S11. **Determination of photosynthesis.** (a)The photosynthesis of transgenic lines in different areas. (b)The photosynthesis of Campus station transgenic lines in same day, but different period (morning, noon and afternoon).

Fig. S12. **The phenotypic traits of transgenic lines and null plants.** (a) Diagram of field trial in different areas. Wuhan (China), Ezhou (China) and Campus station (China). (b) Box plot showing the plant height distribution and (c) was seed morphology for stationary 10 grains cottonseed.

Fig. S13. **The sequence length (a) and phylogenetic (b) of all DEGs.** Most of DEGs length are less than 1kb. In addition, the DEGs were split into four distinct clusters based on phylogenetic analysis showed that many DEGs had relatively close genetic relationship and possibly divided from the same gene family, even the presence of functional redundancy.

Table S1. Summary of sequence read mapping.

Table S2. Primers used in this study for qRT-PCR.

Table S3. Chlorophyll content in transgenic lines of RNAi *GhPEPC1*.

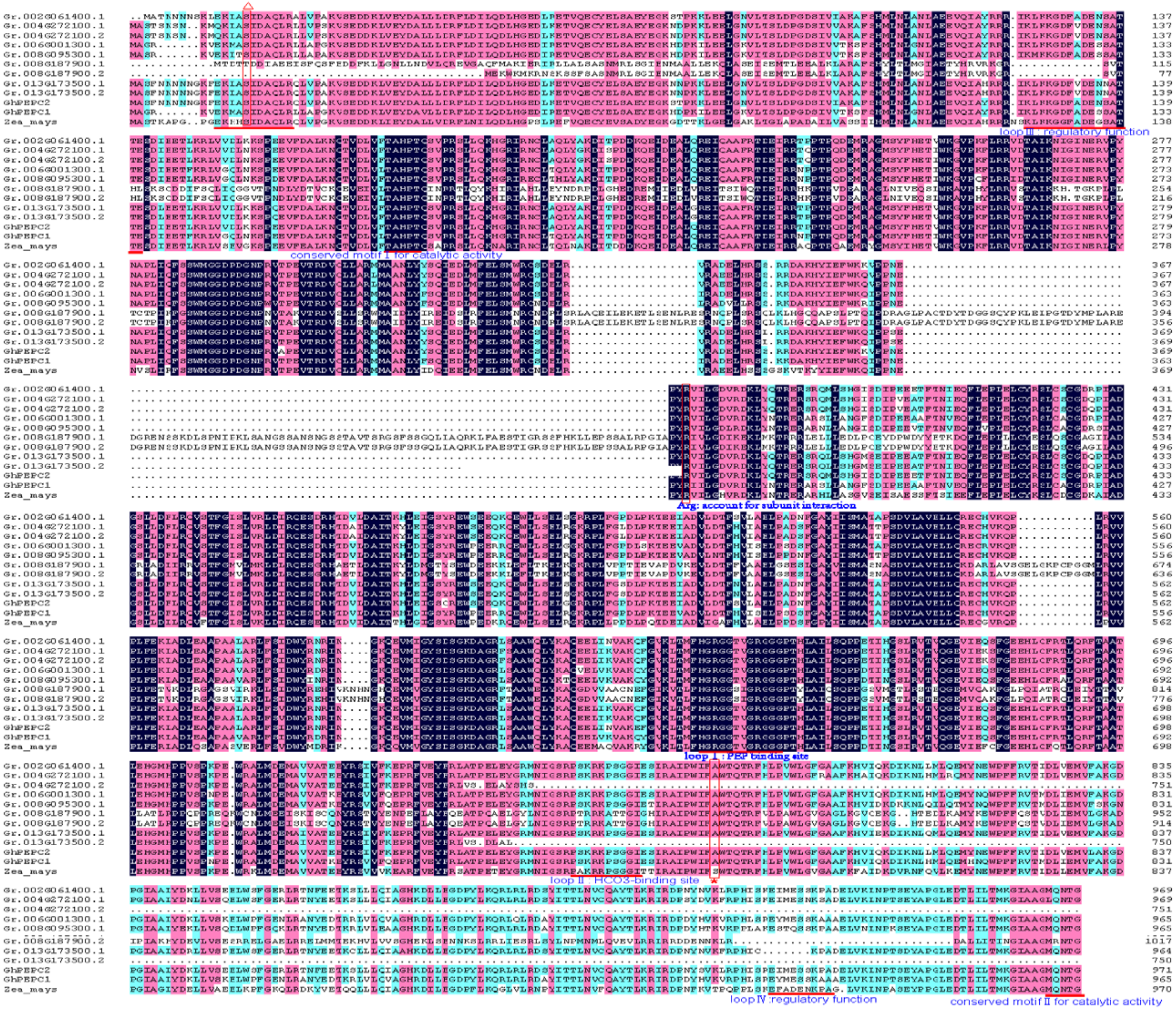
loop III : regulatory function
conserved motif I for catalytic activity
Arg: account for subunit interaction
loop I : PEP binding site
loop II : HCO3-binding site
loop IV :regulatory function
conserved motif II for catalytic activity

Fig. S1.

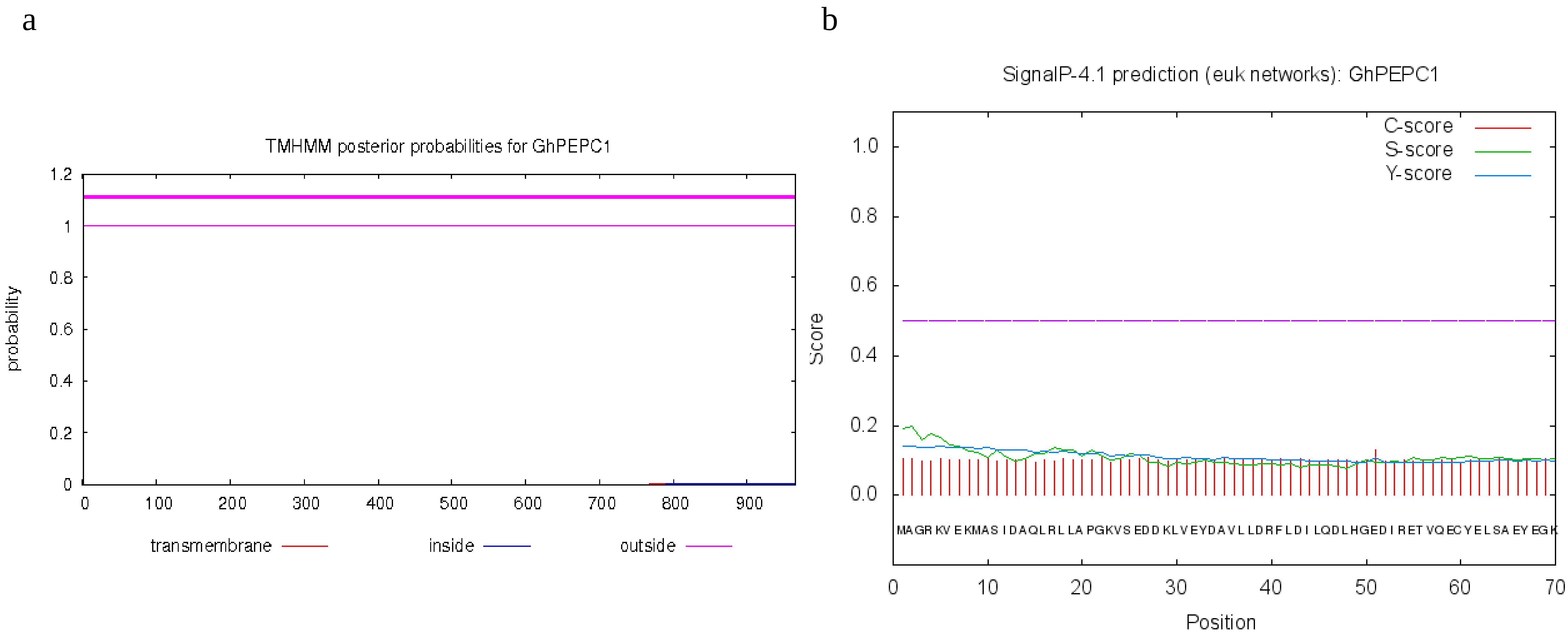
a
TMHMM posterior probabilities for GhPEPC1
probability
1.2
1
0.8
0.6
0.4
0.2
0
0 100 200 300 400 500 600 700 800 900
transmembrane
inside
outside
b
SignalP-4.1 prediction (euk networks): GhPEPC1
C-score
S-score
Y-score
Score
1.0
0.8
0.6
0.4
0.2
0.0
MAGRKVEKMASIDAQLRLLAPGKVSEDDKLVEYDAVLLDRFLDILQDLHGEDIRETVQECYELSAEYEGK
0 10 20 30 40 50 60 70
Position

Fig. S2.

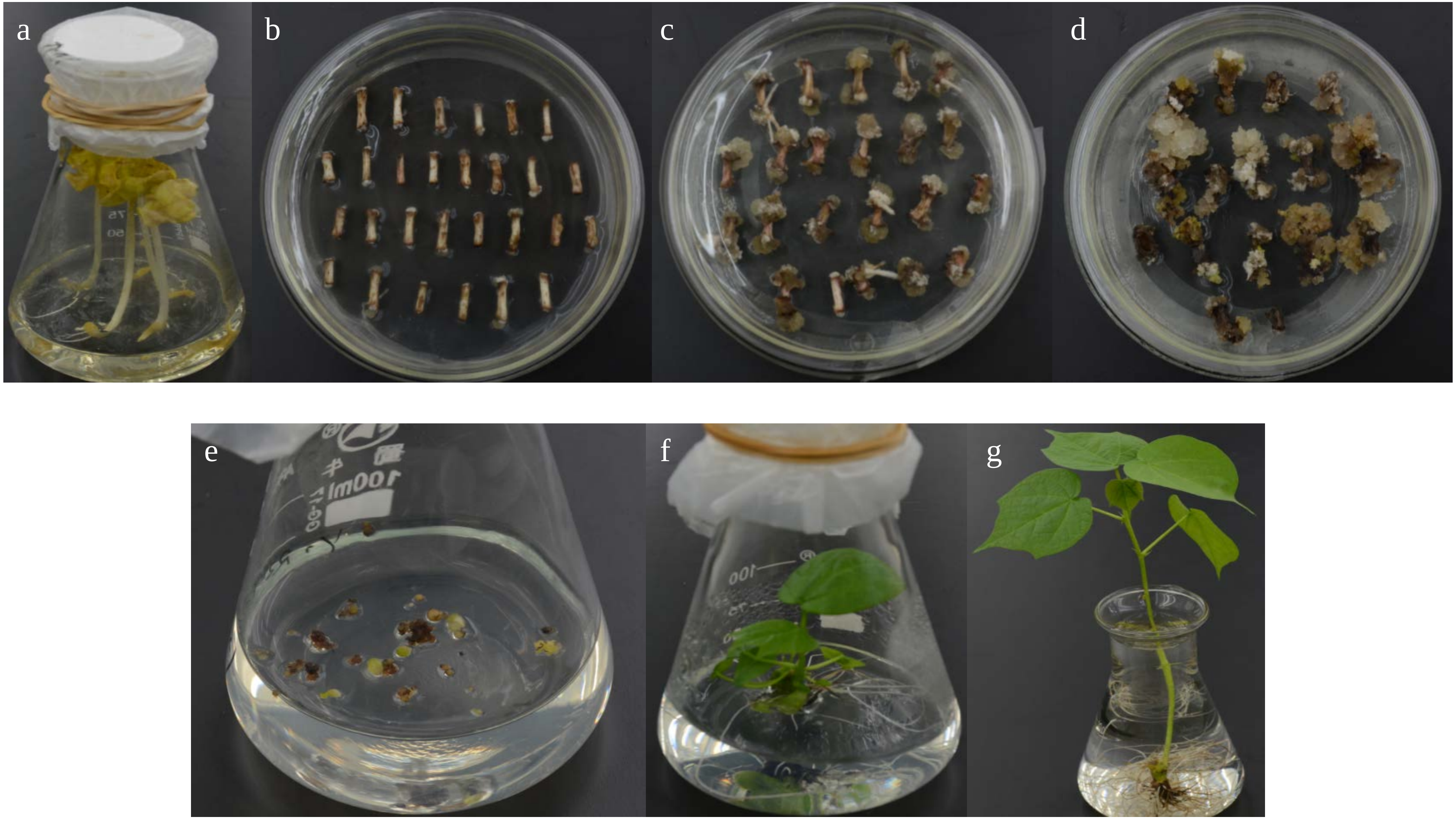

Fig. S3.

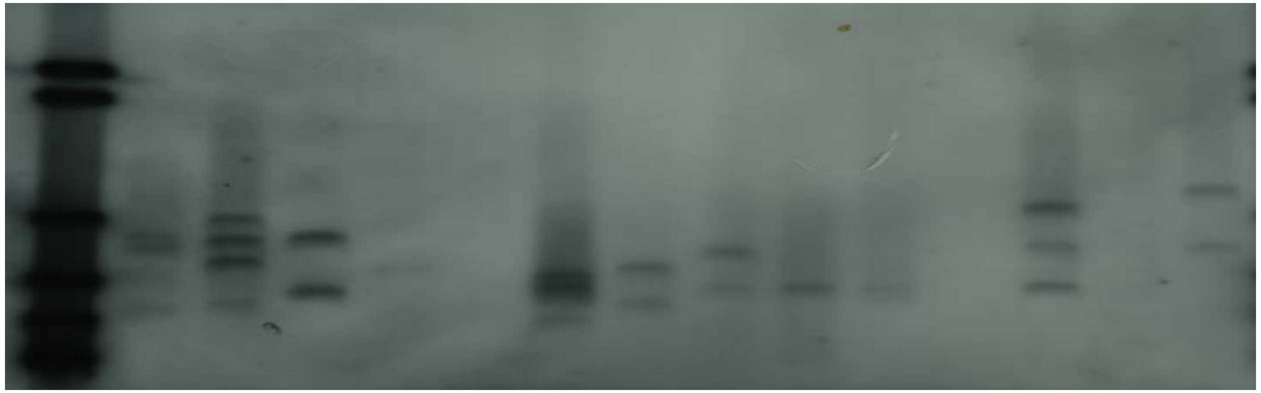

GhPPC M 18 16 32 25 4 3 33 1 8 2 5 20 10 23

Fig. S4.

a

b

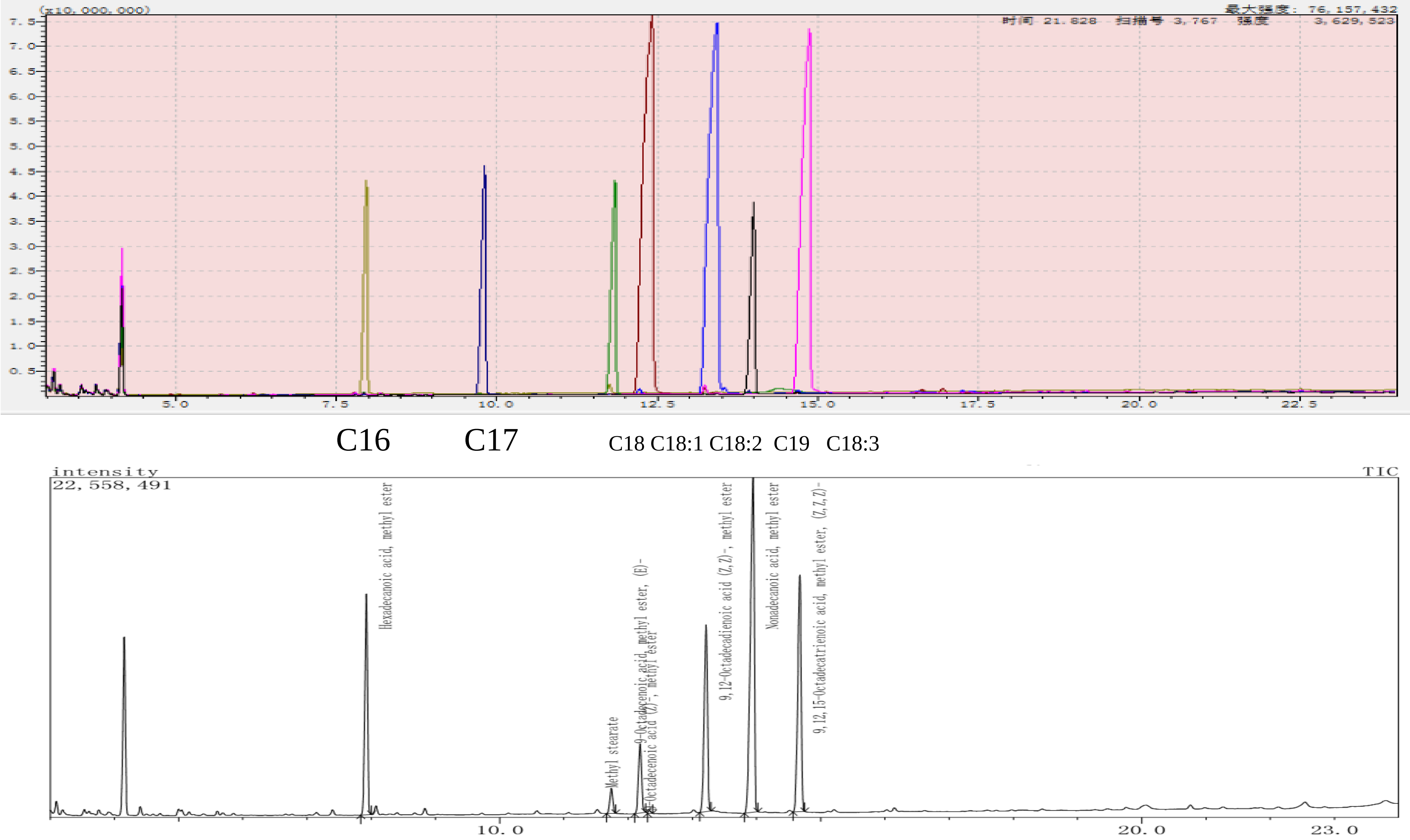
(x10,000,000)
最大强度: 76,157,432
时间 21.828 扫描号 3,767 强度 3,629,523
C16
C17
C18 C18:1 C18:2 C19 C18:3
intensity
22,558,491
TIC
Hexadecanoic acid, methyl ester
Methyl stearate
9-Octadecenoic acid, methyl ester, (E)-
9-Octadecenoic acid (Z)-, methyl ester
9,12-Octadecadienoic acid (Z,Z)-, methyl ester
Nonadecanoic acid, methyl ester
9,12,15-Octadecatrienoic acid, methyl ester, (Z,Z,Z)-

Fig. S5.

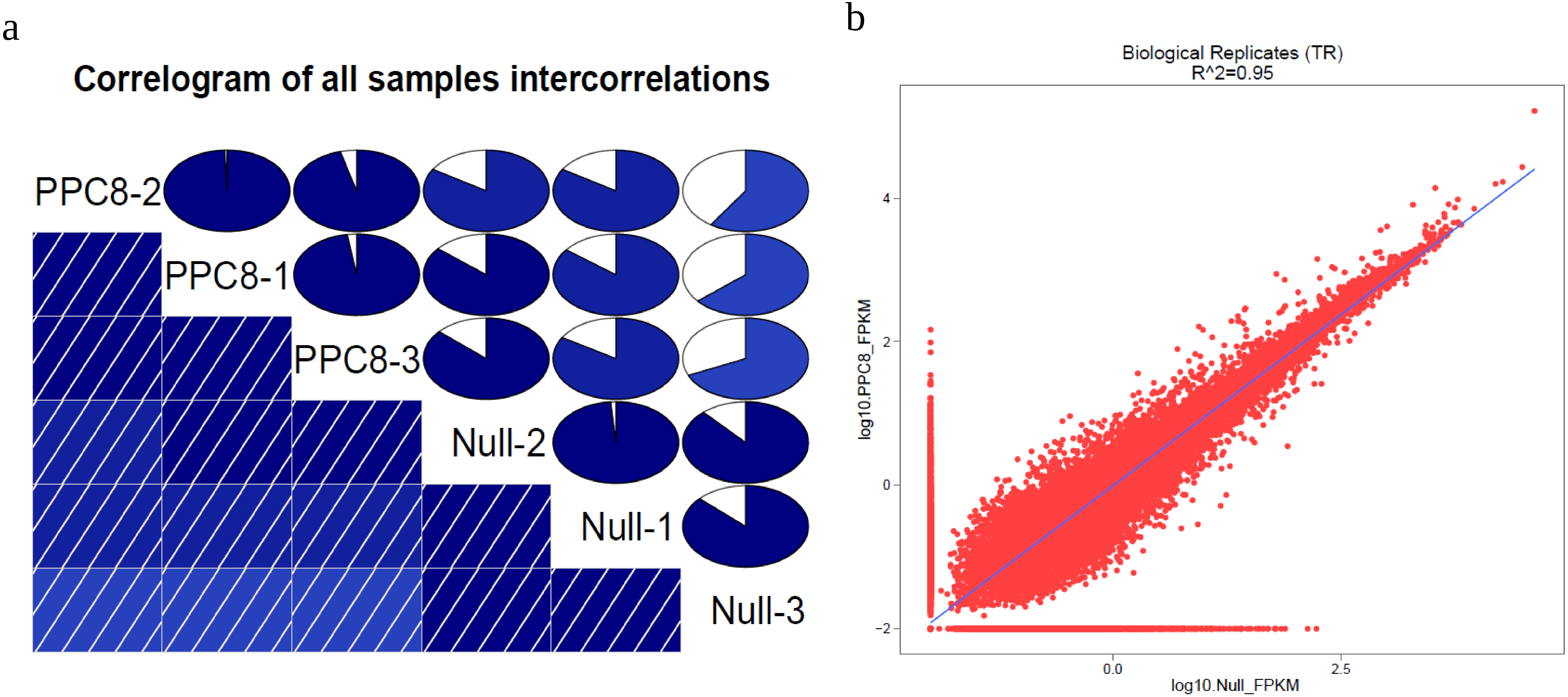
a
Correlogram of all samples intercorrelations
PPC8-2
PPC8-1
PPC8-3
Null-2
Null-1
Null-3
b
Biological Replicates (TR)
R^2=0.95
log10.PPC8_FPKM
4
2
0
-2
0.0
2.5
log10.Null_FPKM

Fig. S6.

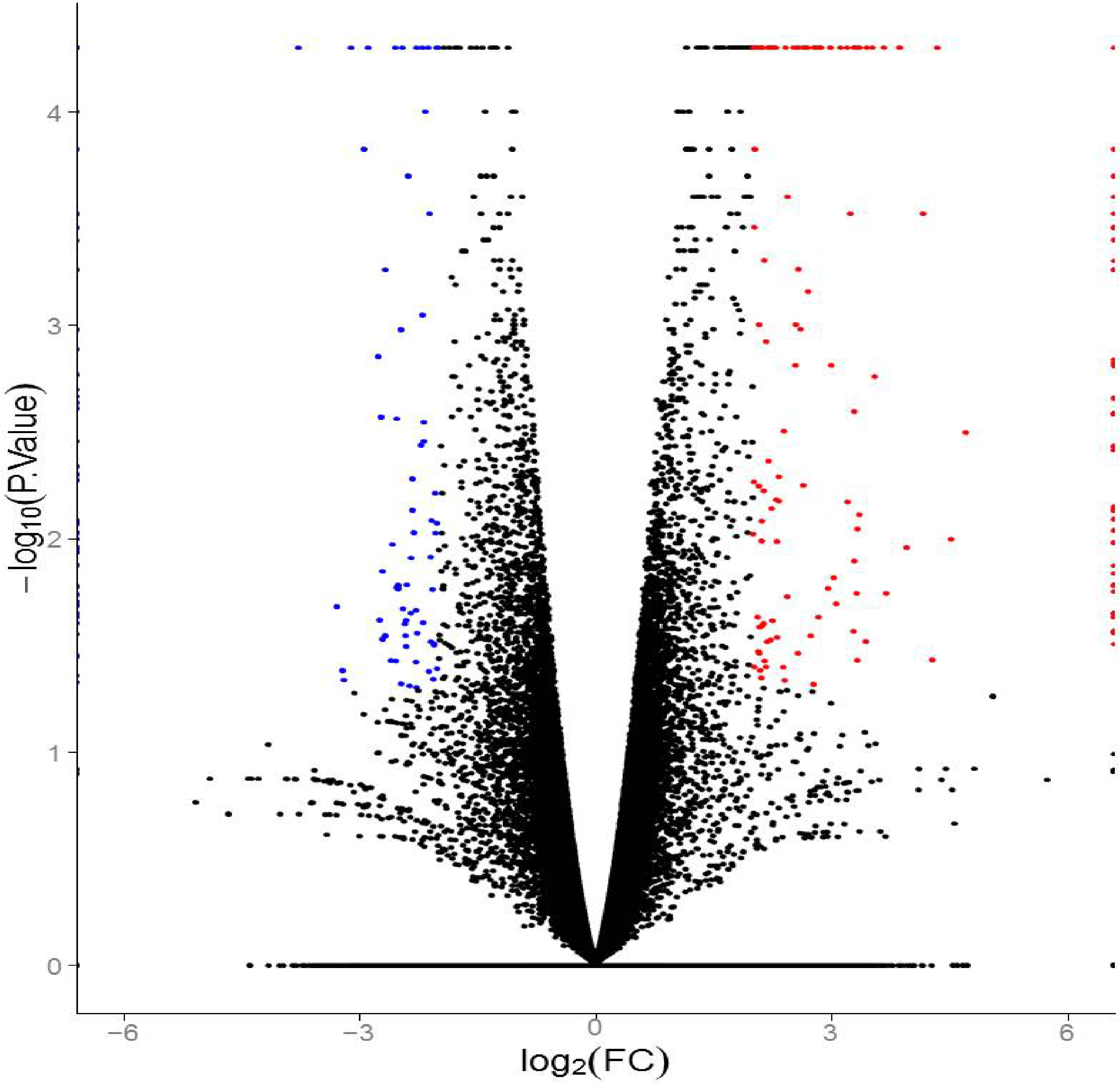

Fig. S7.

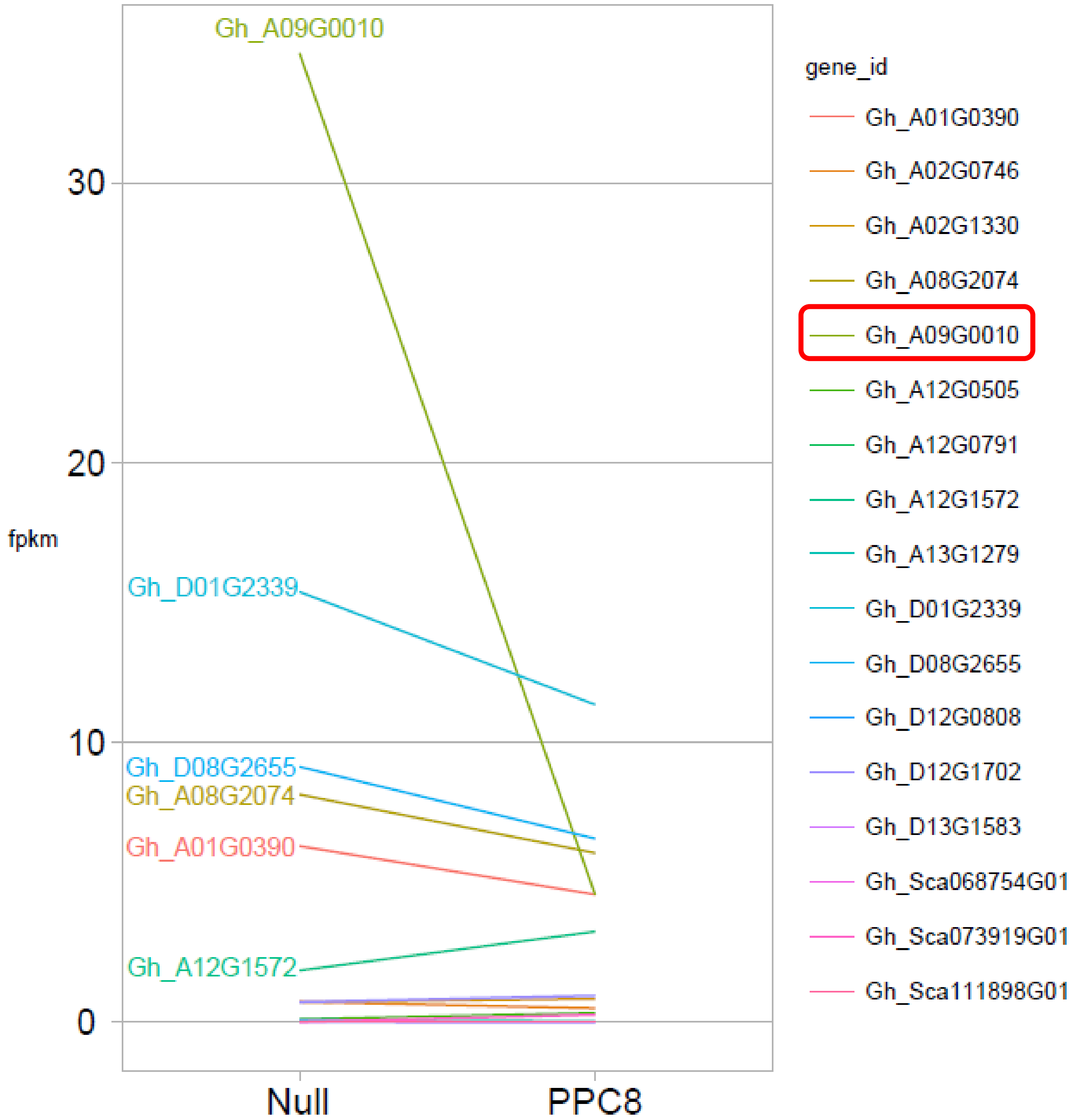

Gh_A09G0010
30
20
10
0
fpkm
Gh_D01G2339
Gh_D08G2655
Gh_A08G2074
Gh_A01G0390
Gh_A12G1572
Null
PPC8
gene_id
Gh_A01G0390
Gh_A02G0746
Gh_A02G1330
Gh_A08G2074
Gh_A09G0010
Gh_A12G0505
Gh_A12G0791
Gh_A12G1572
Gh_A13G1279
Gh_D01G2339
Gh_D08G2655
Gh_D12G0808
Gh_D12G1702
Gh_D13G1583
Gh_Sca068754G01
Gh_Sca073919G01
Gh_Sca111898G01

Fig. S8.

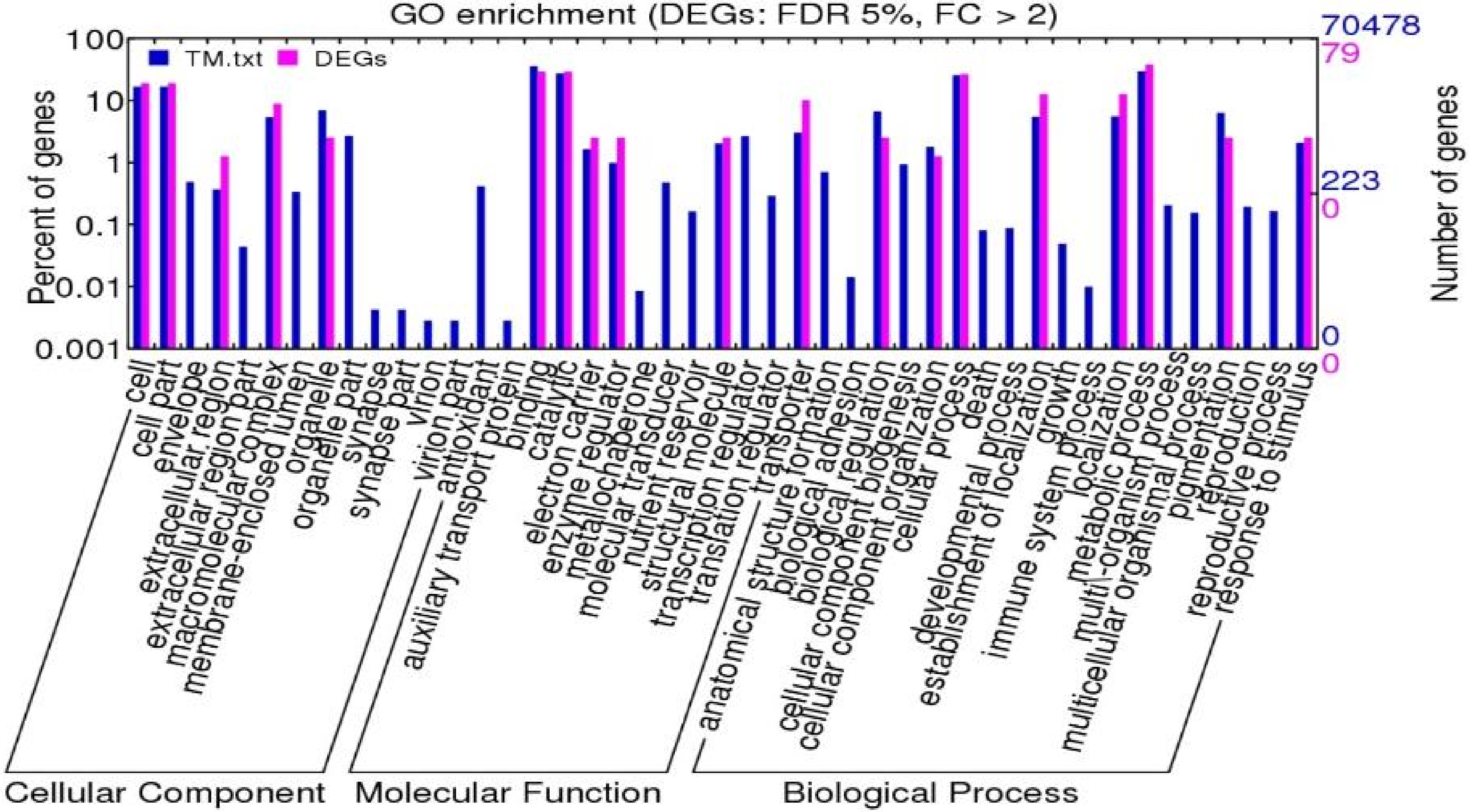
GO enrichment (DEGs: FDR 5%, FC > 2)
TM.txt
DEGs
Percent of genes
Number of genes
100
10
1
0.1
0.01
0.001
70478
79
223
0
0
0
cell
cell part
envelope
extracellular region
extracellular region part
macromolecular complex
membrane-enclosed lumen
organelle
organelle part
synapse
synapse part
virion
virion part
antioxidant
auxiliary transport protein
binding
catalytic
electron carrier
enzyme regulator
metallochaperone
molecular transducer
nutrient reservoir
structural molecule
transcription regulator
translation regulator
transporter
anatomical structure formation
biological adhesion
biological regulation
cellular component biogenesis
cellular component organization
cellular process
death
developmental process
establishment of localization
growth
immune system process
localization
metabolic process
multi-organism process
multicellular organismal process
pigmentation
reproduction
reproductive process
response to stimulus
Cellular Component
Molecular Function
Biological Process

Fig. S9.

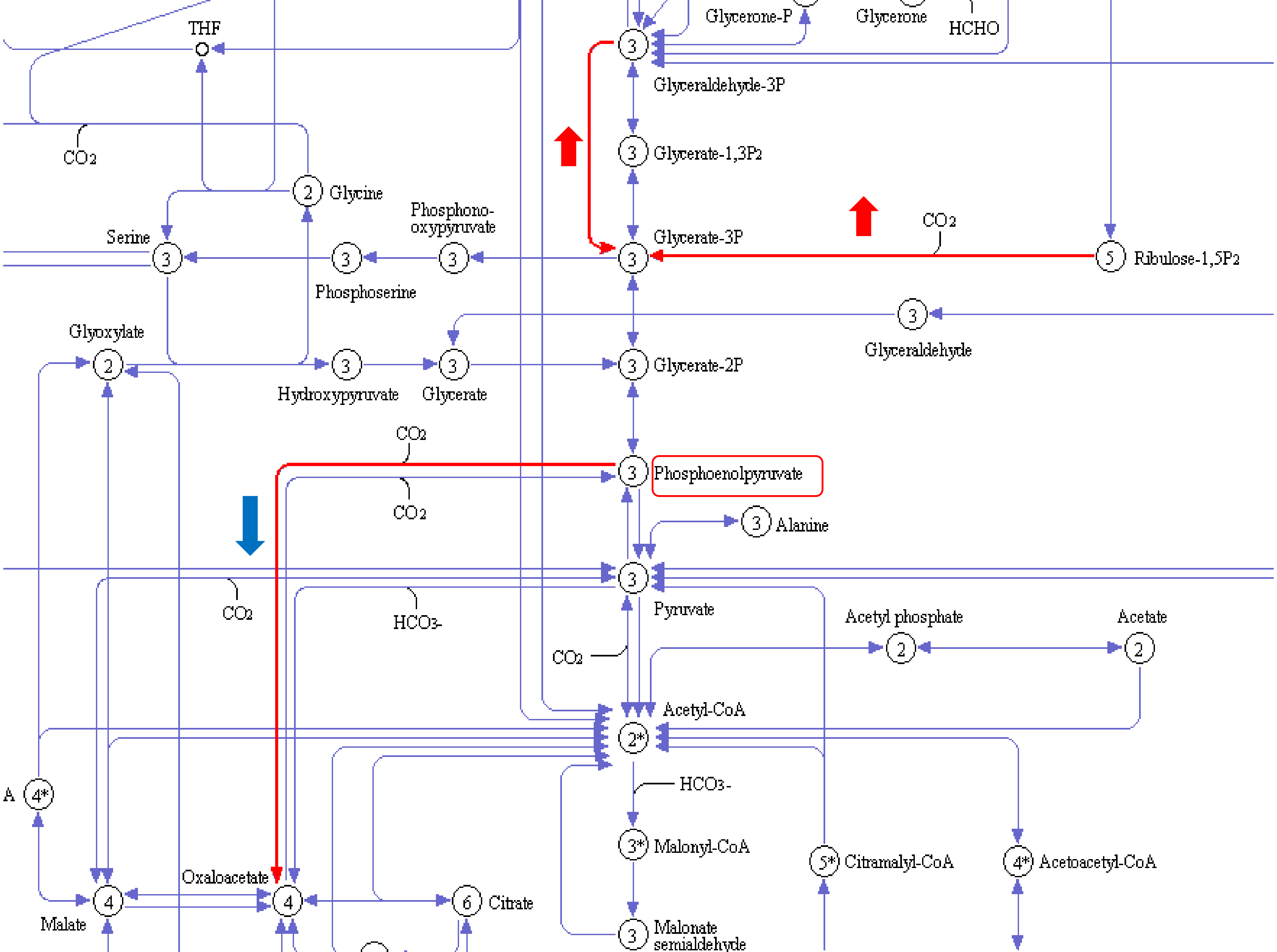
THF
Glycerone-P
Glycerone
HCHO
Glyceraldehyde-3P
CO2
Glycerate-1,3P2
Glycine
Phosphono-
oxypyruvate
Serine
Glycerate-3P
CO2
Ribulose-1,5P2
Phosphoserine
Glyceraldehyde
Glyoxylate
Hydroxypyruvate
Glycerate
Glycerate-2P
CO2
Phosphoenolpyruvate
CO2
Alanine
Pyruvate
CO2
HCO3-
Acetyl phosphate
Acetate
CO2
Acetyl-CoA
HCO3-
A
Malonyl-CoA
Citramalyl-CoA
Acetoacetyl-CoA
Oxaloacetate
Malate
Citrate
Malonate
semialdehyde

Fig. S10.

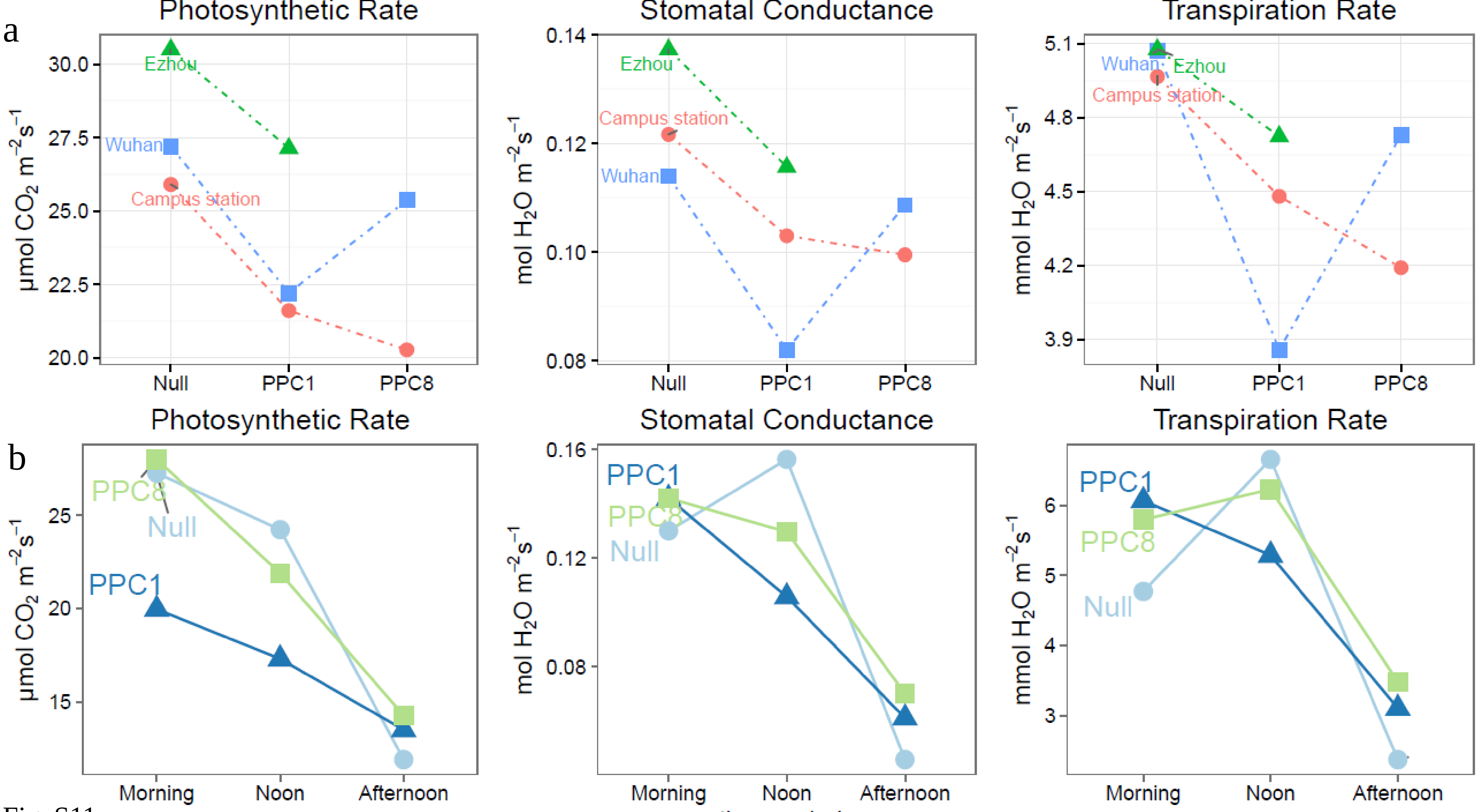

a
Photosynthetic Rate
Stomatal Conductance
Transpiration Rate
μmol CO2 m-2s-1
mol H2O m-2s-1
mmol H2O m-2s-1
30.0
27.5
25.0
22.5
20.0
0.14
0.12
0.10
0.08
5.1
4.8
4.5
4.2
3.9
Ezhou
Wuhan
Campus station
Null
PPC1
PPC8
b
Photosynthetic Rate
Stomatal Conductance
Transpiration Rate
25
20
15
0.16
0.12
0.08
6
5
4
3
PPC8
Null
PPC1
Morning
Noon
Afternoon
time periods

Fig. S11.

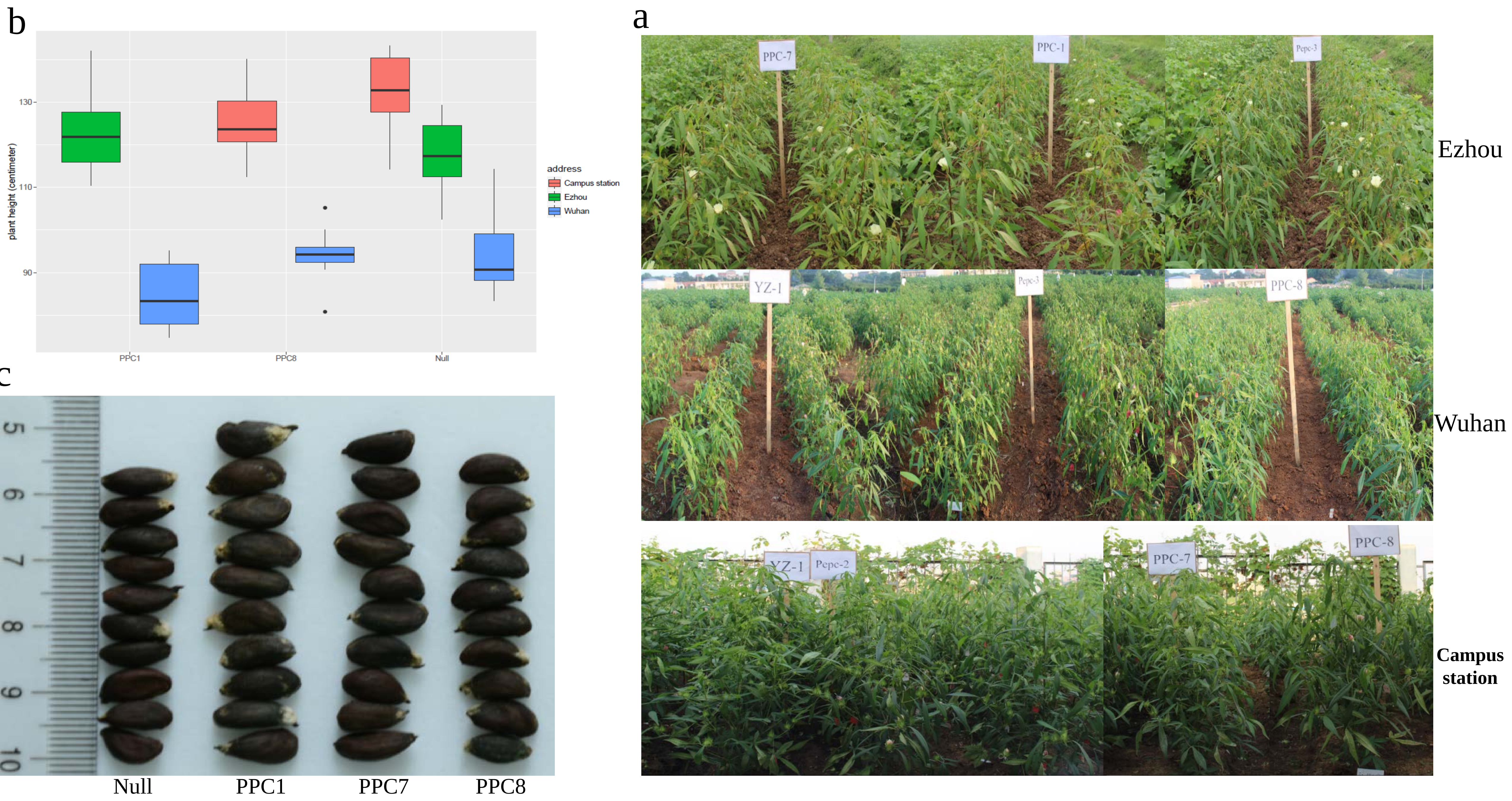
a
PPC-7
PPC-1
Pepc-3
Ezhou
YZ-1
Pepc-3
PPC-8
Wuhan
YZ-1
Pepc-2
PPC-7
PPC-8
Campus station
b
plant height (centimeter)
130
110
90
PPC1
PPC8
Null
address
Campus station
Ezhou
Wuhan
c
5
6
7
8
9
10
Null
PPC1
PPC7
PPC8

Fig. S12.

a

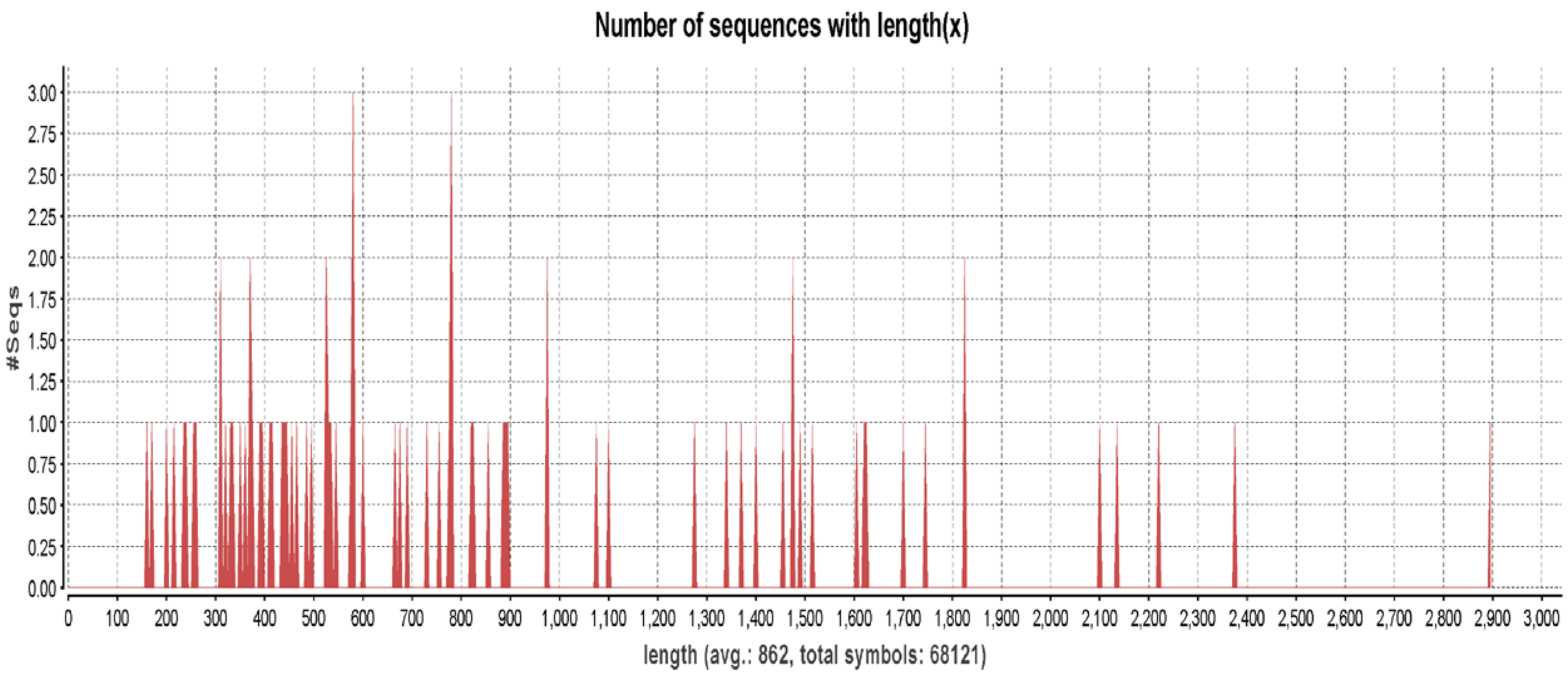
Number of sequences with length(x)
#Seqs
3.00
2.75
2.50
2.25
2.00
1.75
1.50
1.25
1.00
0.75
0.50
0.25
0.00
0 100 200 300 400 500 600 700 800 900 1,000 1,100 1,200 1,300 1,400 1,500 1,600 1,700 1,800 1,900 2,000 2,100 2,200 2,300 2,400 2,500 2,600 2,700 2,800 2,900 3,000
length (avg.: 862, total symbols: 68121)

b

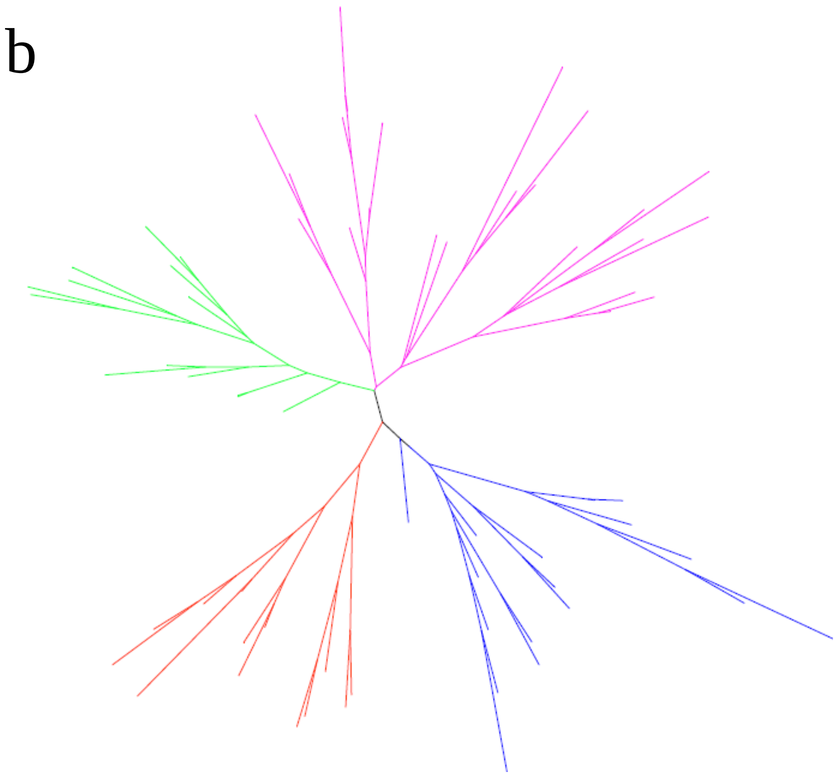

Fig. S13.

Table S1. Summary of sequence read mapping

Sample	Reads	Clean Reads	Mapped reads	Mapped Percent	ultiple Mappec reads	Multiple Mapped Percent	Pair Alignment Rate
Null-1	Reads_1	42310240	18658023	88.20%	2983161	16.00%	77.30%
	Reads_2		17742882	83.90%	2740325	15.40%	
Null-2	Reads_1	37065352	16456898	88.80%	2751402	16.70%	77.60%
	Reads_2		15568456	84.00%	2510196	16.10%	
Null-3	Reads_1	36017112	15700687	87.20%	2701833	17.20%	77.00%
	Reads_2		15023946	83.40%	2512054	16.70%	
PPC8-1	Reads_1	28632034	12751889	89.10%	3469177	27.20%	74.60%
	Reads_2		12031429	84.00%	3155625	26.00%	
PPC8-2	Reads_1	34142702	15283226	89.50%	3645873	23.90%	76.20%
	Reads_2		14494268	84.90%	3343077	23.10%	
PPC8-3	Reads_1	35370716	15743603	89.00%	4188521	26.60%	75.50%
	Reads_2		14970131	84.60%	3860822	25.80%	

Table S2. Primers used in this study for qRT-PCR

Gene	**Primer Sequence**
GhACC-F	5' CAGGCTTGATGTTTGGAGGGA 3'
GhACC-R	3' TTTGGAAGTGCCTTTCGTTGAA 5'
GhPEPC1-F	5' GGCTCCGACTTCGTGATGCTTA 3'
GhPEPC1-R	3' TGGGATGTTCGCTCATACGAGGA 5'

Table S3. Chlorophyll content in transgenic lines of RNAi *GhPEPC1*

Lines \ Address	Campus station			Wuhan		
	Mean(SPAD)	SD	P-value α=0.05	Mean(SPAD)	SD	P-value α=0.05
PPC1	40.89	0.4	**	40.55	0.68	*
PPC8	47.89	1.34	-	39.97	0.35	*
Null	49.09	0.35	-	46.29	0.95	-
